# Covalent organic frameworks for cancer immunotherapy: mechanisms, applications, and prospects

**DOI:** 10.3389/fimmu.2025.1719005

**Published:** 2026-01-08

**Authors:** Yutao Zou, Min Chen, Jiayi Chen, Weiqi Wang, Xiaohua Zheng

**Affiliations:** 1 The People’s Hospital of Danyang, Affiliated Danyang Hospital of Nantong University, Danyang, China; 2 School of Pharmacy, Nantong University, Nantong, Jiangsu, China

**Keywords:** cancer immunotherapy, covalent organic frameworks, immunogenic cell death, immunosuppressive tumor microenvironment, photodynamic therapy

## Abstract

Covalent organic frameworks (COFs) have emerged as promising candidates in cancer immunotherapy, owing to their tunable pore structures, versatile functionality, and favorable biocompatibility. This review systematically highlights recent advances in COF-based materials that enhance immunotherapeutic efficacy through multiple strategies. Particular emphasis is placed on functionalized COFs for remodeling the immunosuppressive tumor microenvironment, by alleviating hypoxia and depleting glutathione, and their role as core sensitizers in various therapeutic modalities, including photodynamic, sonodynamic, radiotherapy, and chemodynamic therapy, to efficiently trigger immunogenic cell death (ICD). In addition, we comprehensively summarize how strategic structural engineering enhances phototherapeutic efficacy. This includes modulating the metal ions incorporated into the COF, controlling COF stacking modes, and adjusting the planarity or conformational twist of the building units to precisely tune bandgap energy and light absorption properties, thereby promoting stronger ICD induction. Furthermore, COFs serve as intelligent delivery platforms capable of controlled release of immune adjuvants and checkpoint inhibitors. The discussion also extends to cutting-edge applications, such as imaging-guided therapy, induction of tertiary lymphoid structure (TLS) formation, and activation of abscopal effects. These developments discussed in this review underscore the immense potential of COFs as multifunctional nanoplatforms in advancing effective and precise combination cancer immunotherapy. The insights provided in this review offer valuable reference for the biomedical applications of COFs, particularly in the integrated development of multimodal therapies and immunotherapy.

## 1 Introduction

Cancer remains one of the most significant global public health challenges (1–3). Over time, treatment strategies have evolved from conventional approaches-such as surgery, chemotherapy, and radiotherapy-to more precise targeted therapies (4–9). In recent years, the emergence of cancer immunotherapy has dramatically reshaped the oncology landscape, offering renewed hope to patients (10–16). Unlike traditional methods that directly target tumor cells, immunotherapy aims to activate or strengthen the body’s own immune system to recognize, attack, and eliminate cancer cells (17–20). Among the most impactful advances is immune checkpoint blockade (ICB), including anti-PD-1/PD-L1 and anti-CTLA-4 antibodies, which have achieved remarkable long-term remission in various advanced cancers, highlighting their substantial clinical promise (21–25). Chemotherapy and radiotherapy are associated with well-known limitations, including drug resistance, severe side effects, and high risks of recurrence and metastasis. In contrast, immunotherapy stands out due to its mechanism-driven specificity, the potential for long-lasting protection through immune memory, and its ability to address tumor heterogeneity (26–30). As a result, it is increasingly becoming a cornerstone of comprehensive cancer treatment.

Despite its promising potential, the broad clinical application of immunotherapy still faces significant hurdles (31–34). A major obstacle is the presence of “cold” tumors, which are characterized by an immunosuppressive tumor microenvironment (TME) and a lack of T-cell infiltration. These features lead to poor response rates to immune checkpoint blockade (ICB), often below 30% (35–40). This suppressive TME arises from a combination of factors, including hypoxia (41–45), overexpression of reactive oxygen species (ROS)-scavenging molecules like glutathione (GSH) (46–49), infiltration of immunosuppressive cells such as regulatory T cells (Tregs) (50–54), and myeloid-derived suppressor cells (MDSCs) (55–58), and inherently low tumor immunogenicity (59–64). Moreover, the therapeutic efficacy of cancer treatments is often limited by systemic toxicity of drugs (65–67), low efficiency in targeted delivery (68–72), and physical barriers presented by solid tumors (73–78). Research has shown that monotherapy with immune-based approaches frequently fails due to factors such as the immunosuppressive TME and insufficient immune activation (79–82). Therefore, developing combination strategies that integrate multiple therapeutic modalities with immunotherapy has become a key approach to improving antitumor outcomes. Numerous studies have demonstrated that therapies such as radiotherapy (RT) (83–86), photodynamic therapy (PDT) (87–93), and photothermal therapy (PTT) (94–99) can effectively induce ICD, leading to the release of tumor-associated antigens. When combined with immune adjuvants that synergistically activate the innate immune system, these treatments hold great promise for triggering robust and long-lasting adaptive immune responses (100–103). In this context, designing and constructing integrated nanoplatforms that combine RT, phototherapy, or sonodynamic therapy (SDT) with the ability to actively remodel the TME, efficiently induce ICD, and enable smart delivery of immunomodulators has emerged as a highly promising research direction (104–109). Such multifunctional nanosystems not only facilitate multimodal synergistic therapy but also offer the potential to transform immunologically “cold” tumors into “hot” tumors that are more responsive to immunotherapy. As a result, these advanced platforms could significantly enhance the overall effectiveness of cancer immunotherapy.

The rise of nanomaterials has provided powerful tools to address the challenges outlined above (110–112). Due to their unique size effects, tunable surface properties, and favorable biocompatibility, nanocarriers offer significant advantages in targeted drug delivery, controlled release, and imaging-guided multimodal therapy (113–116). In the field of immunotherapy, nanoplatforms can co-deliver ICD inducers (e.g., photosensitizers), immune adjuvants (such as CpG, imiquimod or Poly(I:C)), and checkpoint inhibitors within a single system, enabling synergistic delivery and spatiotemporally controlled release (117–120). This approach not only enhances local tumor suppression but also promotes systemic antitumor immunity (121–123). Among various nanomaterials, COFs have recently attracted growing attention. These are crystalline, porous polymers composed of lightweight elements linked by strong covalent bonds (124–127). COFs stand out with exceptional structural designability, high surface area, uniform and adjustable pore sizes, excellent stability, and good biocompatibility, surpassing many conventional nanomaterials (128–131).

COFs are a class of crystalline, porous organic materials composed of light elements such as carbon, hydrogen, boron, oxygen, and nitrogen (132, 133). These frameworks are constructed by linking molecular building blocks through strong covalent bonds in a periodic, ordered arrangement (134). A variety of synthetic strategies have been developed for COF preparation, including solvothermal synthesis, ionothermal methods, microwave-assisted heating, and surface-mediated growth (125, 135). What sets COFs apart is their reliance on reversible covalent chemistry: during synthesis, dynamic bond formation allows for error correction through repeated bond breaking and reformation, ultimately leading to highly ordered structures (136). The resulting frameworks often feature extended π-conjugation, which not only enhances structural rigidity but also imparts remarkable chemical stability (137–139). This combination of structural precision, reversibility-driven crystallinity, and tunable functionality makes COFs uniquely suited for rational design and targeted applications.

COFs have shown significant advantages in the field of anticancer immunocombinational therapy (140–143). Their well-defined porous structures facilitate O_2_ diffusion into the material and efficient release of ROS outward, thereby enhancing ROS generation during PDT and SDT (144–150). This improved ROS activity strengthens interactions with biomacromolecules and promotes ICD, a key mechanism for immune activation. The structural diversity of COFs allows for performance customization by selecting functional building blocks, such as porphyrin-based compounds (151–154). For instance, porphyrin units can coordinate with metal ions like Cu^2+^ to mimic the activities of catalase (CAT) and glutathione peroxidase (GPx), helping to modulate the immunosuppressive tumor microenvironment (TME) (155). Additionally, coordination with Pt^2+^ endows COFs with combined RT and radiodynamic therapy (RDT) capabilities, significantly boosting ICD effects (156). Furthermore, by engineering the topological structure of COFs, materials with mixed planar and twisted configurations can be designed (141). These aggregation-induced emission (AIE)-type COFs exhibit superior phototherapeutic performance and immune activation compared to traditional fully twisted photosensitizers (141). The incorporation of functional linkers containing disulfide bonds, diselenide bonds, or azo bonds enables the construction of stimuli-responsive drug delivery systems that react to high GSH levels or hypoxic conditions, improving targeting and control over therapeutic release (156, 157). Moreover, the high surface area, ordered pore channels, and π-π stacking interactions of COFs allow efficient loading of various therapeutic molecules, making them excellent carriers for multimodal combination therapies (158). Overall, the modular design of COFs enables precise integration of multiple functional components, including photosensitizers (e.g., porphyrins), enzyme-mimicking catalytic sites, and stimuli-responsive linkages, into a single framework. This facilitates the development of “all-in-one” nanoplatforms capable of regulating the TME (e.g., enhancing O_2_ supply and depleting GSH, enabling multi-enhanced therapies (e.g., PDT, SDT, CDT, RT), delivering immune adjuvants, and supporting imaging-guided, integrated immunocombinational cancer treatment (155, 156).

**Figure 1:**
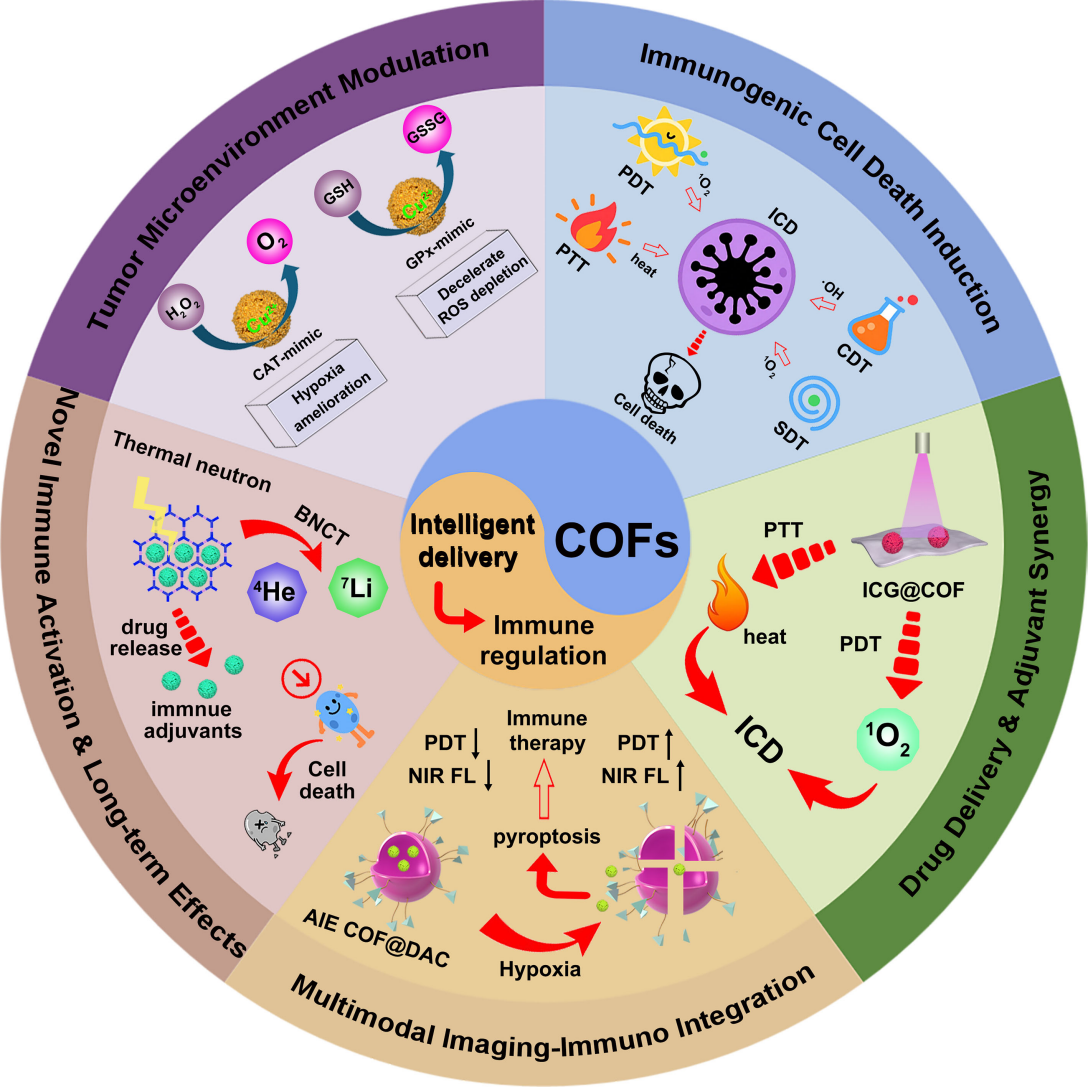
Schematic illustration of COF-based nanomaterials for intelligent drug delivery and immune modulation.

This review provides a systematic summary of recent advances and design strategies in COF-based nanomaterials for enhancing cancer immunotherapy (**Figure 1**). The discussion centers on several key themes. First, it highlights the unique role of COFs in reversing the immunosuppressive tumor microenvironment-through intrinsic enzyme-mimicking activities (such as catalase-like or glutathione peroxidase-like functions) or by carrying functional components-to alleviate hypoxia and deplete GSH (Section 5). Next, it examines how COFs serve as highly efficient sensitizers in photodynamic, sonodynamic, radiotherapy, and chemodynamic treatments, triggering robust ICD and thereby initiating antitumor immune responses (Section 6). The review then explores the application of COFs as smart delivery platforms for immune adjuvants, checkpoint inhibitors, and prodrugs, enabling synergistic immune activation through controlled release (Section 7). Afterwards, this review summarizes the development of COF-based nanoplatforms that integrate diagnostics and therapeutics, offering real-time monitoring and guidance for precise and controllable immunotherapy (Section 8). Finally, it discusses emerging frontiers, including the potential of COFs to induce TLS formation, mediate novel forms of programmed cell death (such as pyroptosis, ferroptosis, and PANoptosis), and integrate diagnostics with therapy in a single platform (Section 9). By synthesizing these developments, this article aims to offer both theoretical insights and practical design principles for next-generation COF-based nanotherapeutics, ultimately supporting their translation into clinical applications.

## 2 Advantages of COF-based nanoplatforms for cancer therapy

Unlike MOFs, COFs are typically constructed entirely from light elements linked by strong covalent bonds, rendering them inherently metal-free and generally free from the risk of toxic metal ion leaching, which contributes to their favorable biocompatibility (159, 160). However, recent advances have enabled the deliberate incorporation of transition metal ions (e.g., Fe, Cu, Pt) into COFs through built-in chelating sites, such as bipyridine and porphyrin moieties, yielding metalated COFs (often denoted as COF-M). In these systems, the coordinated metal centers play crucial mechanistic roles in applications such as phototherapeutic and immunomodulatory applications. Thus, while conventional COFs are metal-free by design, strategically engineered COFs may intentionally integrate metal ions to impart specific functionalities. Compared with liposomes and polymeric nanoparticles, COFs possess highly ordered porous architectures and significantly larger surface areas, which enable substantially higher drug loading capacities and more precise release kinetics (160). Moreover, in contrast to most inorganic nanomaterials, COFs, which are composed solely of light elements, exhibit enhanced biodegradability and reduced concerns regarding long-term accumulation (160). Critically, the robust covalent bonding in COFs ensures excellent chemical stability, while their tunable pore walls allow for precise post-synthetic functionalization. These features collectively establish COFs as a uniquely programmable platform for the integrated delivery of multimodal therapies, such as PDT, PTT, and CDT, together with immunomodulators, an advantage that remains challenging to achieve simultaneously with other existing nanocarriers.

## 3 COF-based reactor and command center for cancer-immunity cycle

**Figure 2:**
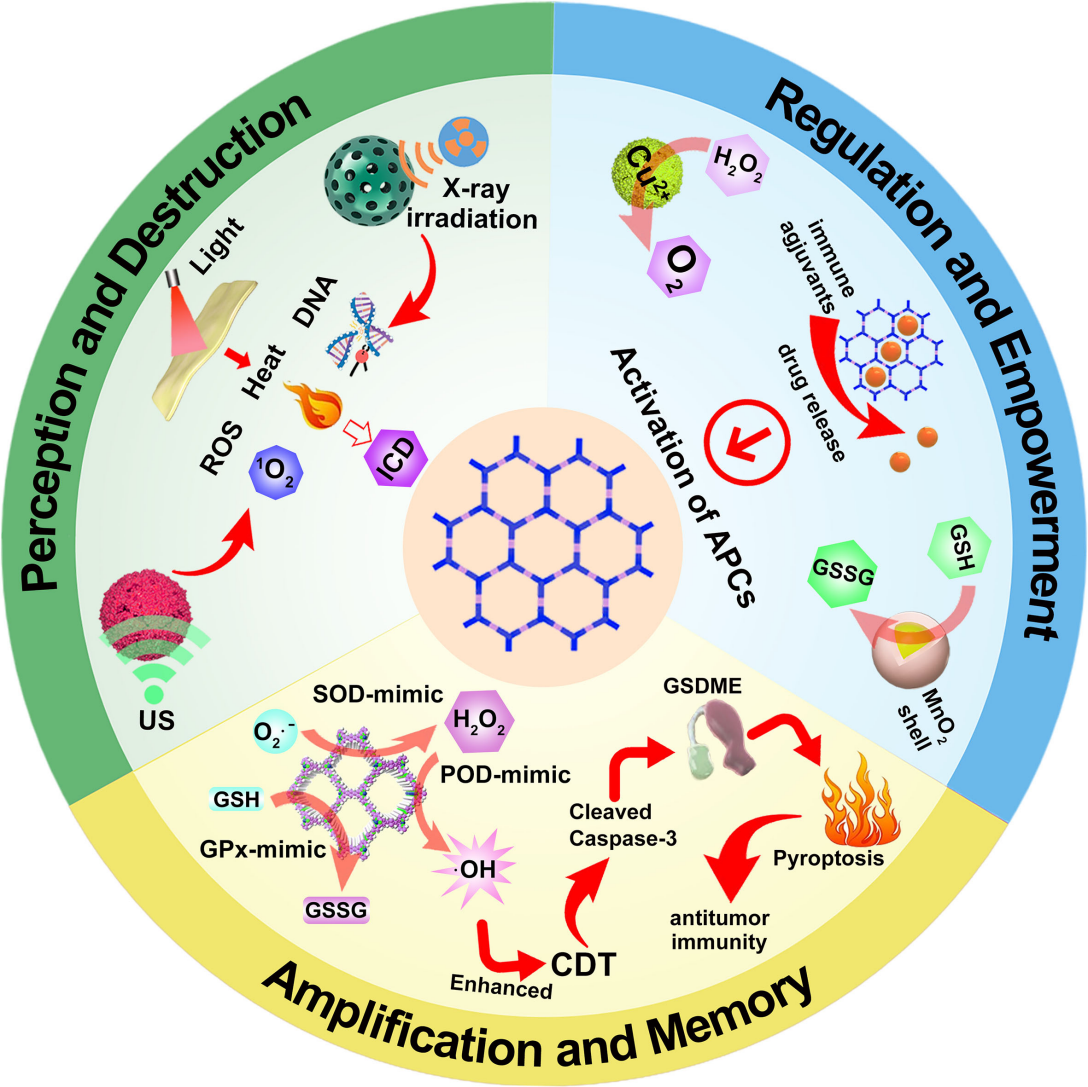
Schematic illustration of COF-based nanomaterials acting as reactors and command centers for intervention and remodeling of the tumor immune cycle.

As cancer immunotherapy research enters a more complex phase, single-mode interventions are increasingly insufficient to overcome the formidable barriers posed by tumor heterogeneity and the immunosuppressive microenvironment (161, 162). Future breakthroughs will depend on intelligent platforms capable of multidimensional and temporally controlled “immune engineering” (163–165). For the first time, this review proposes that COFs have transcended the conventional role of nanocarriers as mere “delivery vehicles,” thanks to their unparalleled structural orderliness and functional programmability. Instead, they now function as active “reactors” and “command centers” capable of dynamically reshaping and directly intervening in the cancer-immunity cycle. Their core mechanism can be conceptualized as an interconnected, self-reinforcing loop. First, “Sensing and Destruction”: COFs, equipped with precisely integrated sensitizing units, efficiently respond to external stimuli (e.g., light, ultrasound, radiation) or endogenous triggers (e.g., H_2_O_2_), generating intense local biochemical effects such as bursts of ROS, hyperthermia, or DNA damage (**Figure 2**) (155, 156, 166). This enables precise tumor cell ablation and robust induction of ICD, leading to tumor antigen exposure. Second, “Modulation and Empowerment”: by leveraging their catalytic properties or delivering immunomodulators through well-defined pores, COFs remodel the tumor microenvironment *in situ* (**Figure 2**). This includes oxygenation, GSH depletion, and localized release of adjuvants or cytokines, thereby removing barriers to dendritic cell activation and facilitating T-cell priming, infiltration, and effector function-essentially fueling the immune response (155, 166, 167). Third, “Amplification and Memory”: through the induction of inflammatory cell death pathways (e.g., pyroptosis, ferroptosis) or the promotion of TLS formation, COFs dramatically amplify the strength and reach of immune signaling (**Figure 2**) (141, 168). This not only eradicates primary tumors but also establishes systemic immune surveillance and long-lasting immunological memory, effectively suppressing distant metastases and recurrence. These three stages do not proceed in a simple linear sequence. Rather, they form a self-amplifying positive feedback loop orchestrated by the COF platform: an improved immune environment enhances treatment efficacy, and stronger therapeutic effects release more immune stimuli. Ultimately, this cycle drives immunosuppressive “cold” tumors toward a self-sustaining “hot” state, tipping them into a trajectory of immune-mediated self-destruction.

**Figure 3:**
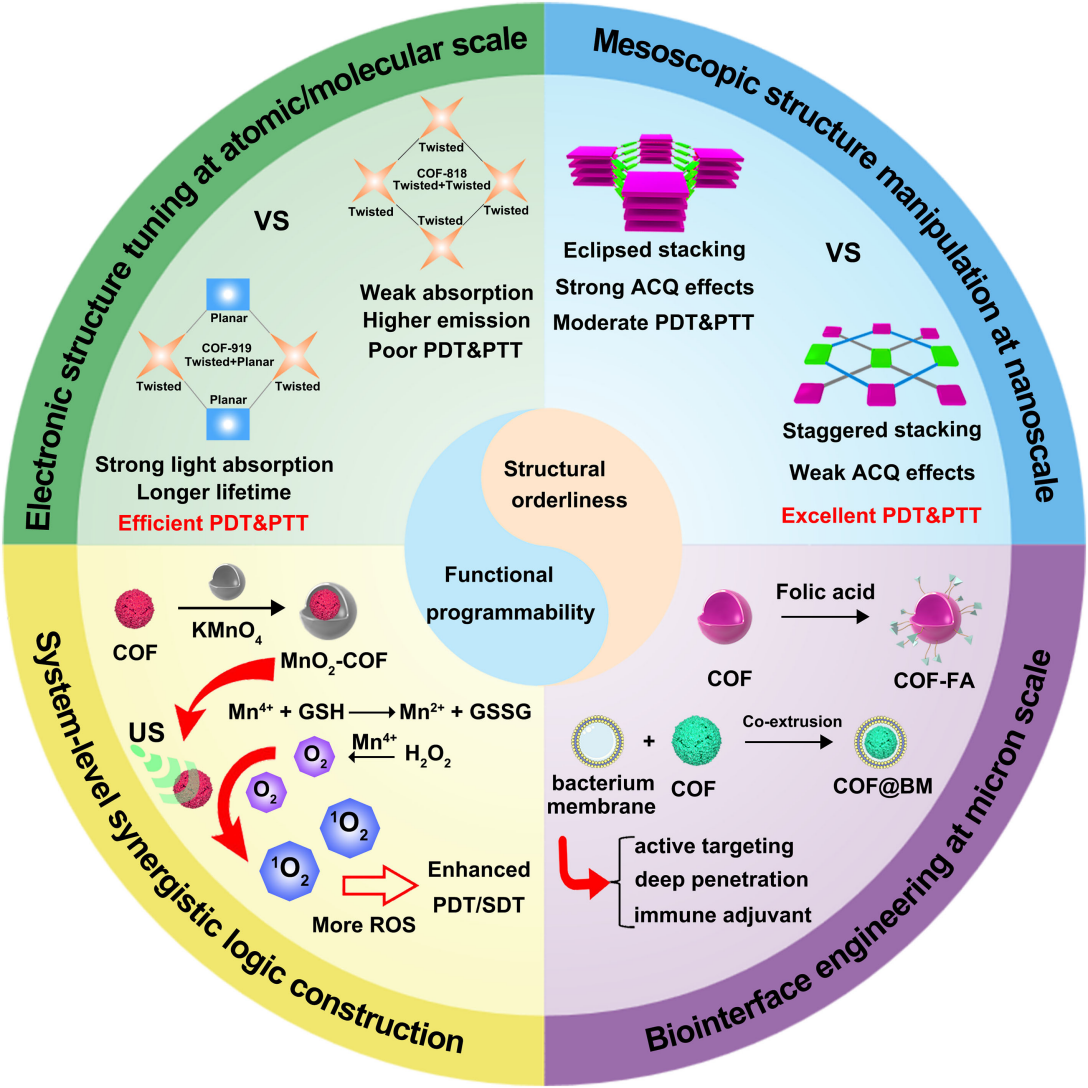
Schematic illustration of the evolution of COF material design from simple structural construction to multi-dimensional functional integration.

To realize this ambitious vision of “immune engineering,” the design philosophy of COF materials must evolve from simple structural construction to sophisticated, multidimensional functional integration. In this context, the review identifies four fundamental design principles, offering a clear roadmap for future research (**Figure 3**). First, by leveraging advanced computational modeling and rational design (**Figure 3**), researchers can optimize the band structure, excited-state lifetime, and electron-hole separation efficiency of COF (141). This fine-tuning is essential to maximize their performance in catalysis, sensitization, and energy transfer-the core of their effectiveness as “reactors” (167). Second, precise engineering of interlayer stacking modes (e.g., staggered vs. eclipsed) (**Figure 3**), pore size distribution, and surface/interface properties enables a balanced optimization of multiple competing demands: exposure of active sites, diffusion of substrates, drug loading capacity, and release kinetics (155). Third, by incorporating biomimetic coatings (such as cell membrane camouflage), targeting ligands (e.g., folic acid), and stimuli-responsive shells (like GSH- or H_2_O_2_-sensitive MnO_2_ layers), COF nanoparticles gain exceptional control over their biological fate (**Figure 3**). These modifications enable prolonged circulation, active tumor targeting, deep tissue penetration, and intelligent, stimulus-triggered disassembly, thereby ensuring precise delivery and accumulation at the tumor site (166, 169, 170). Fourth, this represents the highest tier of design: integrating the above functions according to precise spatiotemporal logic (**Figure 3**). Examples include sequential release systems (e.g., GSH depletion followed by ROS generation), self-sustaining cycles (e.g., catalytic O_2_ production to enhance sonodynamic or photodynamic therapy), or conditionally activated systems (e.g., hypoxia-triggered prodrug activation) (155, 166). Such designs achieve synergistic effects where “1 + 1 > 2”, dramatically improving therapeutic precision and efficacy. Each cutting-edge advancement discussed in this review vividly illustrates the application of these principles. Together, this review outlines a future landscape of cancer immunotherapy driven by COFs: a programmable nanoplatform capable of precisely sensing and intelligently modulating biological processes.

## 4 Multifunctional COF nanoplatforms for antitumor applications

Characterized by high mortality rates, malignant tumors pose a severe threat to global public health and have emerged as a major biomedical challenge (171–175). The interdisciplinary integration of multifunctional nanomaterials and the biomedical field has been proven to hold significant application potential for cancer therapy, offering important possibilities for improving cancer treatment outcomes (176–179). Consequently, the development of effective anticancer therapies has become a focal point of research in both biomedicine and materials science in recent years (180–184). In this context, the exploration of novel nanotherapeutic agents is of paramount importance (185–187). Periodic ordered porous materials, including MOFs and COFs, have been extensively studied in the biomedical field in recent years (188–190). Among these, COFs have garnered significant attention due to their well-defined porous architectures, tunable functionalities, and exceptional structural designability (191). These features enable COFs to serve not only as efficient drug delivery carriers but also as multifunctional platforms capable of synergistically enhancing various therapeutic modalities-such as PDT, PTT, chemodynamic therapy (CDT), SDT-through responsiveness to the tumor microenvironment (e.g., hypoxia, H_2_O_2_, GSH). Moreover, certain COF-based systems can further potentiate antitumor immune responses, enabling combinational immunotherapy. To provide a systematic evaluation of the diverse therapeutic mechanisms mediated by COF-based nanoplatforms, **Table 1** summarizes the morphologies, particle sizes, and underlying action mechanisms of representative COF systems. By systematically comparing structurally diverse COFs in terms of their band structures, light absorption properties, multifunctional therapeutic capabilities, enzyme-mimicking activities, and immunomodulatory mechanisms, this review aims to establish key design principles for optimizing COF-based nanoplatforms. These insights provide a rational framework for tailoring COF architectures to achieve synergistic multimodal therapy and robust immune activation through precise structural and functional engineering.

**Table 1:** Synthesis of various COF-based nanoplatforms for combined cancer therapy integrating multiple treatment modalities with immunotherapy.

Material	Treatment/Immunotherapy		Property and therapeutic advantages	Ref
	Treatment	Immunotherapy		
COF-618-Cu	PDT/PTT	ICD+αPD-1	Size≈150 nm, staggered packing mode, CAT-mimic, GPx-mimic, 660 nm	(155)
MnO_2_-Poly(I:C)@COF	SDT	ICD+ Poly(I:C)	Spherical NPs, Size≈180 nm, CAT-mimic, GPx-mimic, MRI, ultrasound	(166)
COF-909-Ni, COF-909-Fe, COF-909-Cu	PTT/CDT pyroptosis	adaptive immune responses/ αPD-1	Nanoribbon-like nanosheets, GSDME-dependent pyroptosis, SOD-mimic, GPx-mimic, 808 nm	(167)
COF-TATB	PDT/CDT	ICD+αPD-1	Nanosphere, Size≈100 nm, CAT-mimic, GPx-mimic, 635 nm, 10 min	(192)
CFAP	PDT/PTT CDT	ICD+αPD-1	Nanosphere, zeta = −28.7 mV, 650 nm (PDT), 808 nm (PTT)	(193)
PorSe-CuPt	RT-RDT PANoptosis	ICD	diamond-shaped NPs, Size≈100 nm, CBL0137 (Z-DNA formation), CAT-mimic, X-ray irradiation	(156)
COF-606	PDT	ICD+αPD-1	nanosized crystal, diameter≈100 nm, two-photon absorption (808 nm)	(194)
PgP@Fe-COF	SDT/CDT	ICD+αPD-1	NPs, GPx-mimic, ultrasound	(195)
FCCCP	SDT/CDT	ICD+CpG	core-shell NPs, Size≈200 nm, magnetic targeting, ultrasound	(196)
COF-919	PDT/PTT ferroptosis pyroptosis	adaptive immune responses/ αPD-1	GSH depletion, twisted+ planar structure, diameter≈204 nm, 660 nm (PDT), 808 nm (PTT)	(141)
CFe-FA	chemotherapy	ICD+pro-IMQ	Nanosphere, diameter≈45.09 nm, zeta = −4.88 mV, folic acid (targeting), DOX (chemotherapy)	(169)
ZTN@COF@poloxamer	PDT PARPi	ICD	Spherical nanocapsule, diameter≈120 nm, zeta = −11.33 mV, niraparib (inhibit DNA repair), 660 nm	(197)
COF-306@FM	PDT pyroptosis	*F.n.* membrane (immune adjuvant) +αPD-1	diameter≈120 nm, zeta = −17.4 mV, bacterium membrane, 660 nm	(170)
ICG@COF-1@PDA	PDT/PTT	ICD	Rounded nanosheets, lateral size (130–160 nm), ICG@COF-1 (-38.1 mV), 808 nm	(158)
TD@COFs	PDT pyroptosis	ICD	Nanosphere, white light, DAC (pyroptosis), hypoxia-triggered-drug release	(157)
B-COF	BNCT	ICD+imiquimod	Nanosphere (1002.1 ± 121.6 nm, –46.7 ± 4.0 mV), M boron content (20.55 wt%)	(198)
TPDA-ViBT-COF	PDT/PTT	ICD+αCTLA4	Fusiform NPs (187 nm), 10 min, TLS formation, 660 nm	(168)

MRI, magnetic resonance imaging; SOD, superoxide dismutase; RT-RDT, radiotherapy-radiodynamic therapy; pro-IMQ, pro-imiquimod; PARPi, poly-ADP-ribose polymerase (PARP) inhibitors; TLS, tertiary lymphoid structures.

## 5 Enhanced antitumor strategies via COF-mediated remodeling of the tumor microenvironment

**Figure 4:**
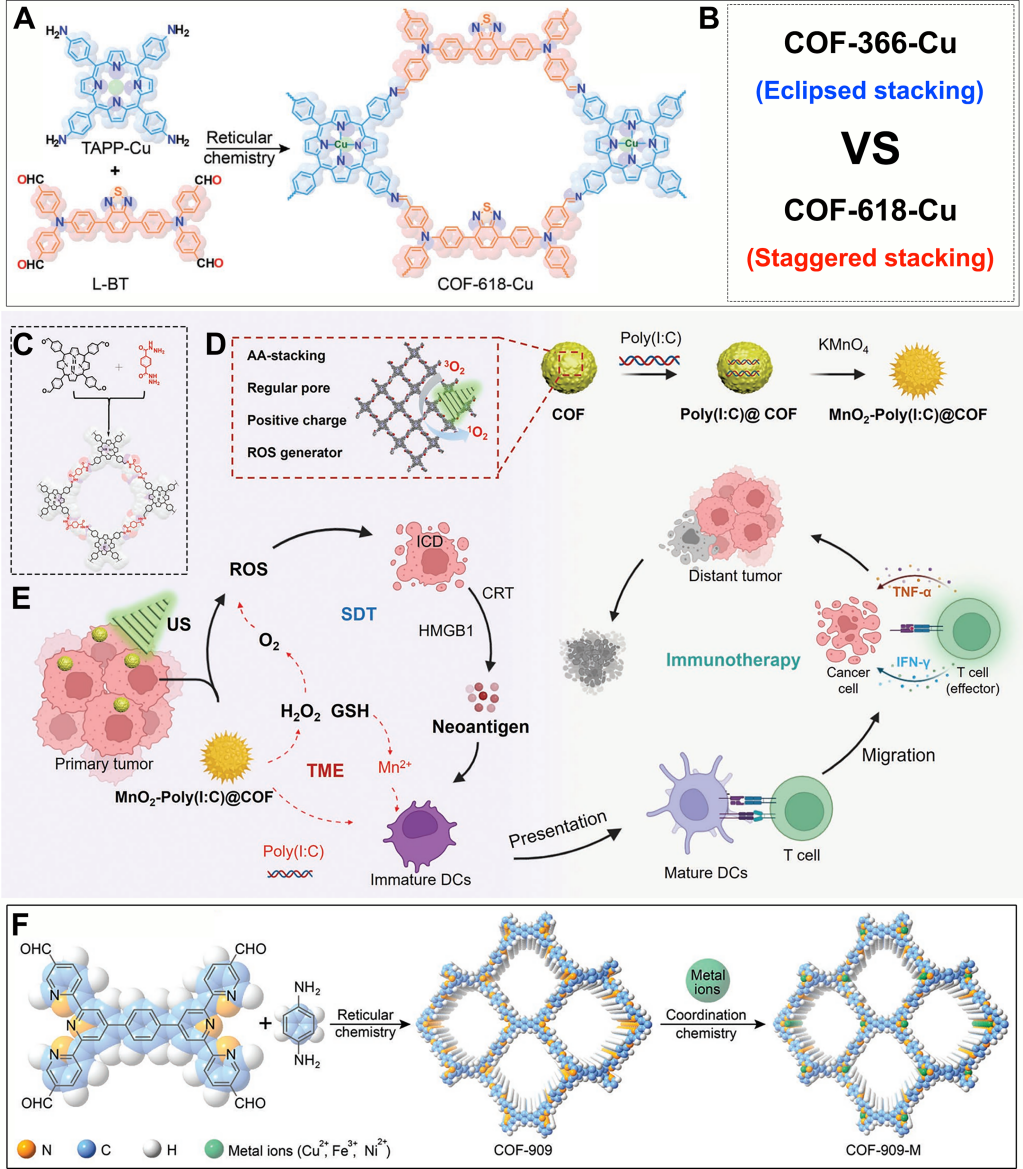
**(A)** Synthesis procedure for COF-618-Cu. **(B)** Stacking mode of COF-618-Cu and COF-366-Cu. Reproduced with permission from Ref (155). Copyright (2022), Wiley-VCH GmbH. **(C)** Synthetic procedure for the porphyrin-based COF. **(D)** Preparation process of MnO_2_-Poly(I:C)@COF composites. **(E)** Schematic illustration of MnO_2_-Poly(I:C)@COF composites for GSH depletion and O_2_ generation-enhanced sonodynamic therapy and combined immunotherapy against tumors. Reproduced with permission from Ref (166). Copyright (2022), Wiley-VCH GmbH. **(F)** Synthesis procedure for the COF-909-M. Reproduced with permission from Ref (167). Copyright (2022), Wiley-VCH GmbH.

The design flexibility of COFs allows them to be precisely engineered with enzyme-mimicking activities, actively reversing the immunosuppressive TME, thereby enhancing antitumor immunotherapy. For instance, Sun et al. developed a multifunctional COF-618-Cu using Cu-coordinated porphyrin molecules and L-BT as monomers (**Figure 4A**) (155). They discovered that the synthesized COF-618-Cu adopts a distinct staggered stacking configuration (**Figure 4B**). For comparative purposes, they also prepared an eclipsed-stacked COF, designated COF-366-Cu (**Figure 4B**). The study demonstrates that this unique interlayer spatial architecture in COF-618-Cu effectively mitigates porphyrin aggregation and quenching, thereby enhancing the generation of hydroxyl radicals (•OH) and superoxide anions (O_2_•^-^). This improved performance is attributed to COF-618-Cu’s lower binding energy and reduced bandgap. In addition, COF-618-Cu displays superior CAT activity (O_2_ production), GPx activity (GSH depletion). Due to these multifunctional advantages, COF-618-Cu achieves enhanced photodynamic and photothermal effects, resulting in a strong ICD for superior antitumor performance. GSH depletion significantly elevates intracellular oxidative stress, which can profoundly influence the progression and therapeutic outcomes of various diseases (199–202). This system’s Cu-618-Cu acts as an efficient “ROS amplifier” by simultaneously alleviating tumor hypoxia and depleting overexpressed GSH through its CAT-like and GPx-like activities, clearing dual obstacles for subsequent therapies.

Building upon successful modulation of critical TME factors, more sophisticated designs aim to integrate multiple functionalities for synergistic effects. For example, Lu et al. prepared a COF-based host material using tetrakis(4-formylphenyl) porphyrin and p-phenylenediacrylic hydrazide as building blocks (**Figure 4C**) (166). Utilizing electrostatic interactions, positively charged COFs were successfully loaded with Poly(I:C), followed by coating MnO_2_ on the surface via an *in-situ* growth strategy, ultimately yielding MnO_2_-Poly(I:C)@COF composites (**Figure 4D**). This nanoplatform not only consumes GSH and produces O_2_ but also enables real-time monitoring of TME reversal via magnetic resonance imaging (MRI), releasing adjuvants and Mn^2+^ to directly activate immune cells, showcasing an integrated diagnostic and therapeutic approach (**Figure 4E**). Specifically, it was revealed that MnO_2_ within the composite exhibits enzyme-like catalytic functions, capable of both reducing intracellular GSH levels and promoting H_2_O_2_ decomposition to produce O_2_ (**Figure 4E**). Both lowering the concentration of reductive molecules in the TME and increasing local O_2_ levels enhance the potential for ROS generation, providing a foundation for improved SDT efficacy and ICD.

Regulation of the TME is not limited to O_2_ and GSH; precise control over H_2_O_2_ metabolic homeostasis is equally crucial. For example, Sun et al. fabricated COF-909 using tetraformaldehyde monomer and diaminobenzene as monomers, further modifying it into metal-containing COF-909-M (Cu^2+^, Fe^3+^, Ni^2+^) via post-modification strategies (**Figure 4F**) (167). The authors discovered that COF-909-M possesses multi-enzyme mimetic functions, particularly acting as an “H_2_O_2_ homeostasis disruptor.” The materials are capable of mimicking superoxide dismutase (SOD) to promote H_2_O_2_ generation. In addition, the Cu^+^ species generated via the Cu^2+^/Cu^+^ redox cycle can act as a peroxidase-mimic (POD-like), catalyzing the conversion of H_2_O_2_ into highly cytotoxic •OH. Notably, COF-909-Cu also displays GPx-like activity by depleting intracellular GSH, thereby suppressing H_2_O_2_ scavenging and significantly elevating intracellular H_2_O_2_ levels. This cascade effect creates a favorable microenvironment for enhanced CDT and effectively triggers pyroptosis. This photothermal effect of the COF-909-M series not only accelerates the Fenton reaction but also synergistically boosts CDT efficacy. Importantly, COF-909-Cu is found to induce GSDME-dependent pyroptosis, highlighting its mechanistic specificity. Moreover, combination therapy using COF-909-Cu and αPD-1 achieves robust therapeutic outcomes, effectively inhibiting the growth of both primary and distant tumors, thus providing a reliable strategy for abscopal tumor suppression. This system fully leverages the diversity of COF structures and preparation methods, presenting new ideas for developing multifunctional pyroptosis inducers. In addition, it demonstrates that active regulation of the TME can achieve enhanced immunotherapy outcomes.

**Figure 5:**
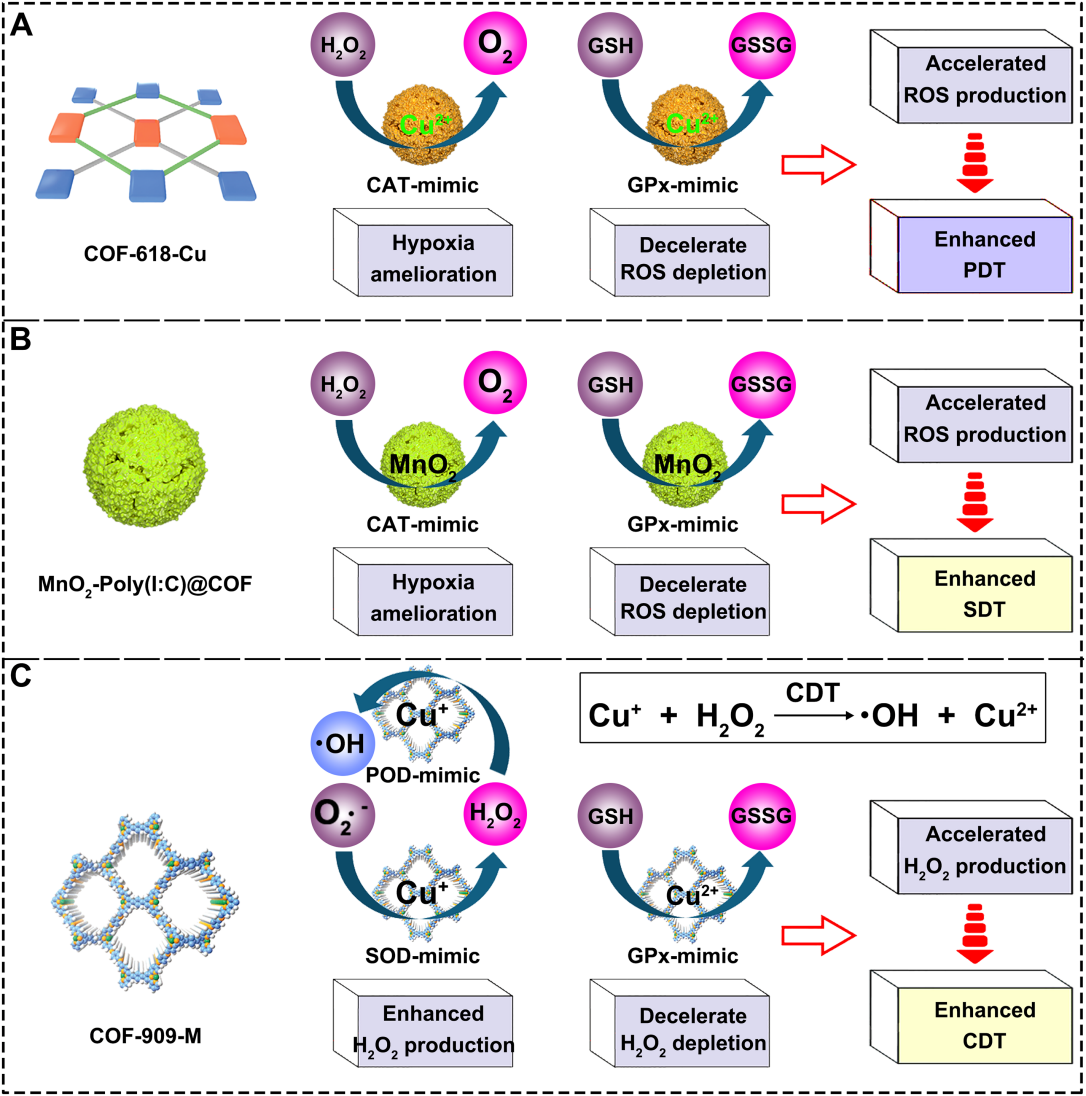
**(A)** Mechanism of COF-618-Cu for GSH depletion and O_2_ generation-enhanced PDT. **(B)** Mechanism of MnO_2_-Poly(I:C)@COF for GSH depletion and O_2_ generation-enhanced SDT. **(C)** Mechanism of COF-909-Cu for GSH depletion and H_2_O_2_ generation-enhanced CDT.

To support their rapid proliferation and invasive metastasis, cancer cells exhibit abnormally high metabolic activity, leading to excessive activation of ROS-producing enzymatic systems, thus generating higher levels of H_2_O_2_ than normal cells (203–206). To counteract oxidative stress from elevated levels of ROS like H_2_O_2_, cancer cells compensate by upregulating GSH synthesis to maintain intracellular redox balance, avoiding oxidative damage. This dynamic, tumor-favorable redox balance established by high concentrations of GSH and H_2_O_2_ in the TME not only inhibits ROS-based therapies but also indirectly causes immune suppression. Recent research has shown that regulating these intratumoral microenvironments (excess H_2_O_2_ and GSH) holds potential for enhancing cancer treatments (207). The three studies highlighted in this section utilize Cu^2+^/Cu^+^ redox reactions to elevate O_2_ levels and lower reductive GSH levels (**Figure 5A**) (155), Mn^4+^/Mn^2+^ redox reactions to increase O_2_ levels and decrease GSH levels (**Figure 5B**) (166), and the cycling between Cu^2+^ and Cu^+^ ions to regulate H_2_O_2_ and GSH levels along with hydroxyl radical concentrations (**Figure 5C**) (167). Through rational design, these studies adjust the immunosuppressive intratumoral microenvironment, paving the way for enhanced PDT, SDT, CDT, and subsequent boosted immune effects.

## 6 COF-based therapeutic modalities for inducing ICD

While functionalized COFs have achieved notable success in reversing the immunosuppressive TME, remodeling the TME itself is not the ultimate goal of therapy but rather a prerequisite for more effective tumor killing. A successfully “heated” TME requires a powerful and precise “trigger” to thoroughly eliminate tumor cells and release their internal antigenic signals. This naturally directs attention to one of COFs’ most promising applications: serving as highly efficient sensitizers that induce ICD through multiple energy conversion pathways, thereby initiating the critical chain of antitumor immune responses.

**Figure 6:**
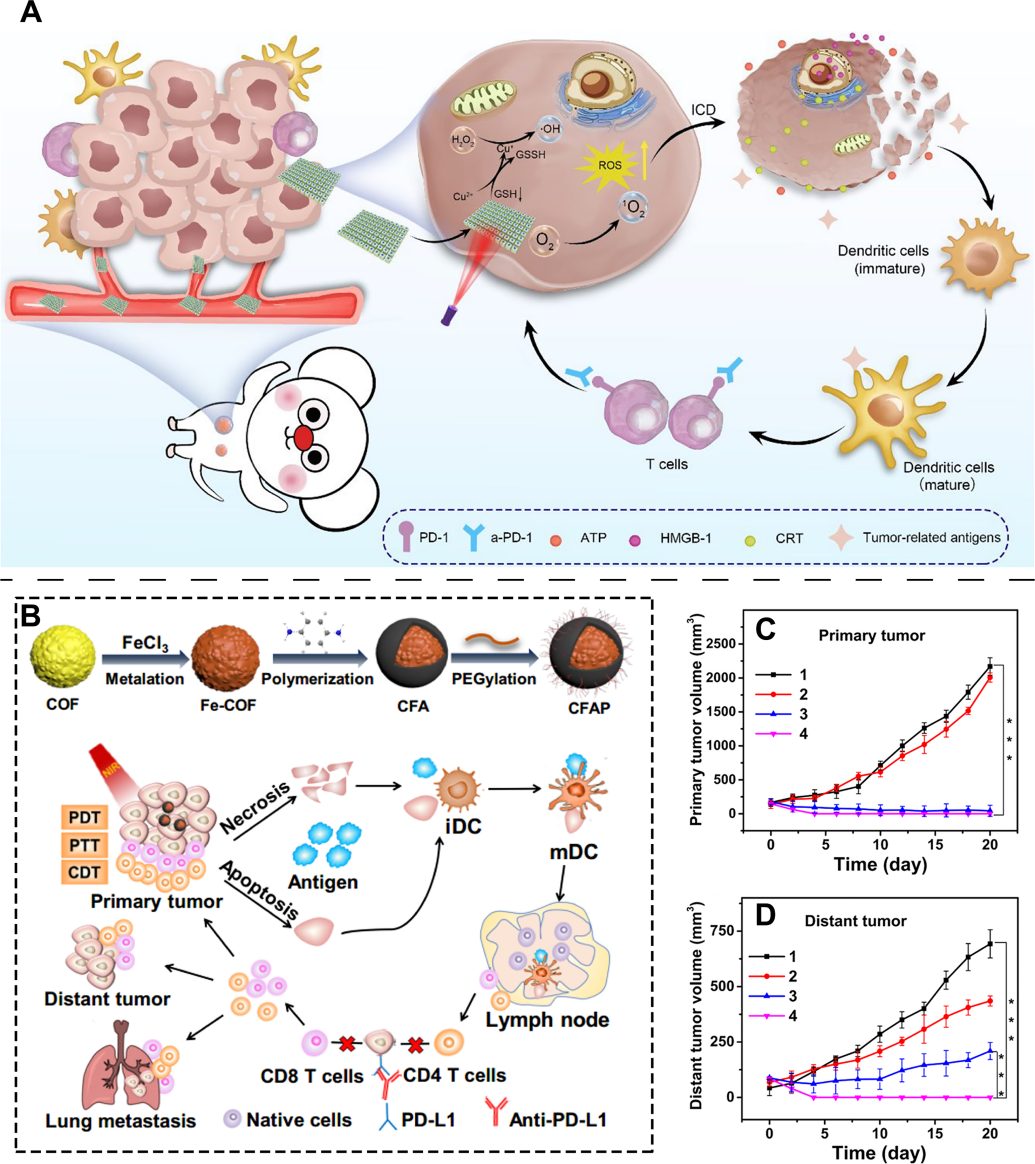
**(A)** Schematic illustration of porphyrin-based COFs for combined PDT and ICD in antitumor treatment. Reproduced with permission from Ref (192).. Copyright (2022), American Chemical Society. **(B)** Synthetic procedure of the CFAP composite and schematic illustration of its combined PDT, PTT, and CDT for tumor suppression. **(C)** Inhibition effect of CFAP on the primary tumor in a bilateral tumor model. **(D)** Inhibition effect of CFAP on the distant tumor in a bilateral tumor model. [(1) PBS; (2) PBS + a-PD-L1; (3) CFAP + 650 + 808 nm; (4) CFAP + 650 + 808 nm + a-PD-L1]. Reproduced with permission from Ref (193).. Copyright (2020), American Chemical Society.

For example, Tang et al. synthesized COF-TATB using porphyrin-based monomers and further prepared Cu@COF-TATB by incorporating Cu^2+^ ions as binding sites (192). The Cu^2+^ ions not only reduce intracellular GSH levels in cancer cells but also react with H_2_O_2_ to generate hydroxyl radicals. Upon light irradiation, the porphyrin units produce ^1^O_2_. The combined ROS effectively trigger the ICD pathway in cancer cells (**Figure 6A**). When combined with anti-PD-1 (aPD-1), this system further amplifies the immune response, offering a novel strategy for cancer therapy. Beyond generating ROS to induce ICD, more sophisticated structural designs can integrate multiple therapeutic modalities into a single COF platform, creating stronger synergistic effects to more effectively “ignite” the immune response. Pang et al. developed a COF using a triamine monomer and terephthalaldehyde, followed by Fe^3+^ metalation, polymerization of p-phenylenediamine, and PEG modification to obtain the multifunctional CFAP material (**Figure 6B**) (193). This platform enables combined PDT, PTT, and CDT, along with their associated antitumor immune effects. As shown in **Figure 6C-D**, CFAP not only effectively suppresses primary tumor growth but also inhibits distant tumors, demonstrating that this multimodal approach efficiently activates the mouse immune system and induces ICD.

**Figure 7:**
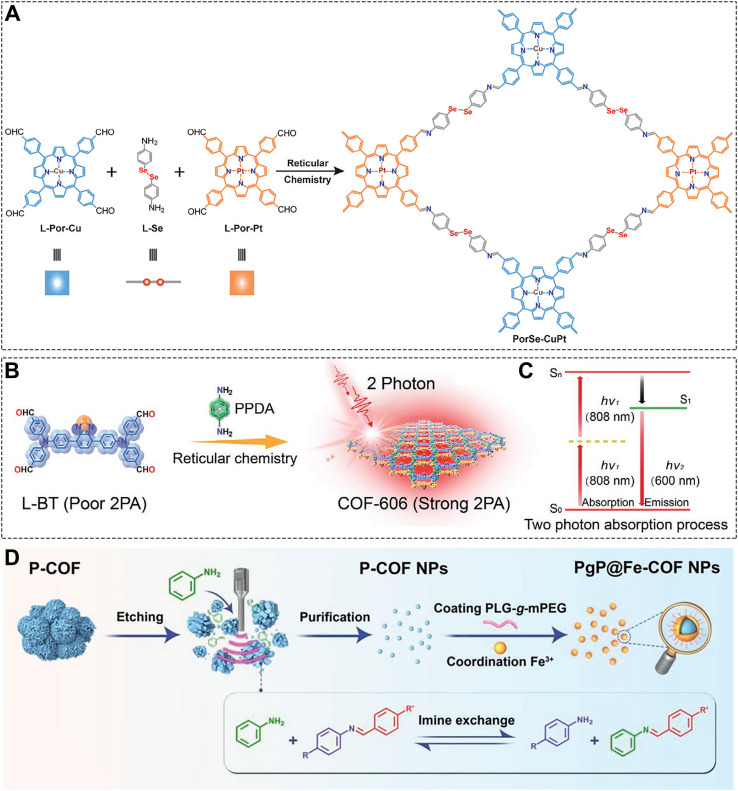
**(A)** Synthetic procedure of the PorSe-CuPt. Reproduced with permission from Ref (156). Copyright (2025), Wiley-VCH GmbH. **(B)** Synthetic procedure of COF-606 and **(C)** its optical mechanism of 2PA absorption. Reproduced with permission from Ref (194). Copyright (2021), Wiley-VCH GmbH. **(D)** Synthesis process of Pg@Fe-COF NPs. Reproduced with permission from Ref (195). Copyright (2022), Wiley-VCH GmbH.

This deep integration of phototherapy with CDT significantly enhances local tumor killing. However, hypoxia and antioxidant defenses within tumors remain major barriers to maximizing ROS production. Thus, COF designs aimed at reversing the immunosuppressive TME have emerged. Deng et al. constructed PorSe-CuPt COF using a Cu-centered porphyrin, a diselenide-linked diamine ligand, and a Pt-centered porphyrin as monomers (**Figure 7A**) (156). Under radiation, the diselenide bonds break, releasing the drug payload. Meanwhile, the Pt component enhances radiation absorption by the COF. The large amount of ROS generated upon irradiation further promotes diselenide bond cleavage and drug release. Concurrently, Cu^2+^ catalyzes O_2_ generation from H_2_O_2_, alleviating hypoxia. The released drug CBL0137, activated by ROS and radiation, inhibits DNA repair and induces Z-DNA formation. The resulting COF@CBL system improves the tumor immune microenvironment, triggers PANoptosis (a form of programmed cell death), and promotes extensive release of tumor antigens, leading to a robust immune response. Radiotherapy is a treatment modality with strong tissue penetration capabilities (208–210). This study employs an X-ray activation strategy, offering a novel approach for immunotherapy based on COF nanoplatforms.

The success of radiotherapy highlights COFs’ potential in treating deep-seated tumors. However, the limited tissue penetration of light remains an inherent challenge for optical therapies. Two-photon (2PA) activation of photosensitizers offers a promising solution to improve tissue penetration. Sun et al. synthesized COF-606 from L-BT and p-phenylenediamine monomers (**Figure 7B**) (194). They demonstrated that COF-606 efficiently absorbs two-photon irradiation (808 nm) and generates ROS effectively (**Figure 7C**). The authors demonstrated that, one-photon excitation (560 nm) allowed imaging to a depth of 130 μm, whereas two-photon excitation (808 nm) achieved a significantly greater penetration depth of 233 μm-clearly demonstrating the superior tissue penetration of two-photon excitation. *In vivo* tumor inhibition studies further confirmed that treatment with COF-606 and 808 nm irradiation potently suppresses tumor growth. Notably, elevated levels of key ICD markers-ATP, calreticulin (CRT), and HMGB1-were observed in the COF-606 + 808 nm group, indicating robust activation of immune responses within the tumor microenvironment and supporting the synergistic effect of PDT and immunotherapy. Overall, this work presents a rationally designed COF with strong 2PA absorption at 808 nm, offering an effective strategy for targeting deep-seated tumors. Intriguingly, while the monomer L-BT exhibited poor photostability under 808 nm light, its stability was markedly enhanced upon incorporation into the COF structure. This improved stability likely arises from the extended π-π conjugation in the COF structure, which protects the active units-providing a promising approach to stabilize otherwise photolabile organic molecules for biomedical applications.

Light and sound represent two key external energy sources, each with distinct advantages in tissue penetration. When light faces depth limitations, ultrasound offers a promising alternative. Even for photosensitizers located within deep-seated lesions, the sonodynamic activation approach can still efficiently promote the conversion of oxygen into singlet oxygen. Furthermore, this technique circumvents phototoxicity issues, completely eliminating the risk of cutaneous photosensitivity reactions during treatment. Based on this, Tian et al. fabricated P-COF using tetraaminoporphyrin as a monomer, followed by etching, purification, Fe^3+^ coordination, and polymer coating to obtain Pg@Fe-COF NPs (**Figure 7D**) (195). The Fe^3+^ ions endow the COF with GSH-depleting and CDT capabilities, enhancing SDT efficacy (**Figure 7D**). Combining SDT with anti-PD-L1 antibody therapy led to even greater therapeutic outcomes.

**Figure 8:**
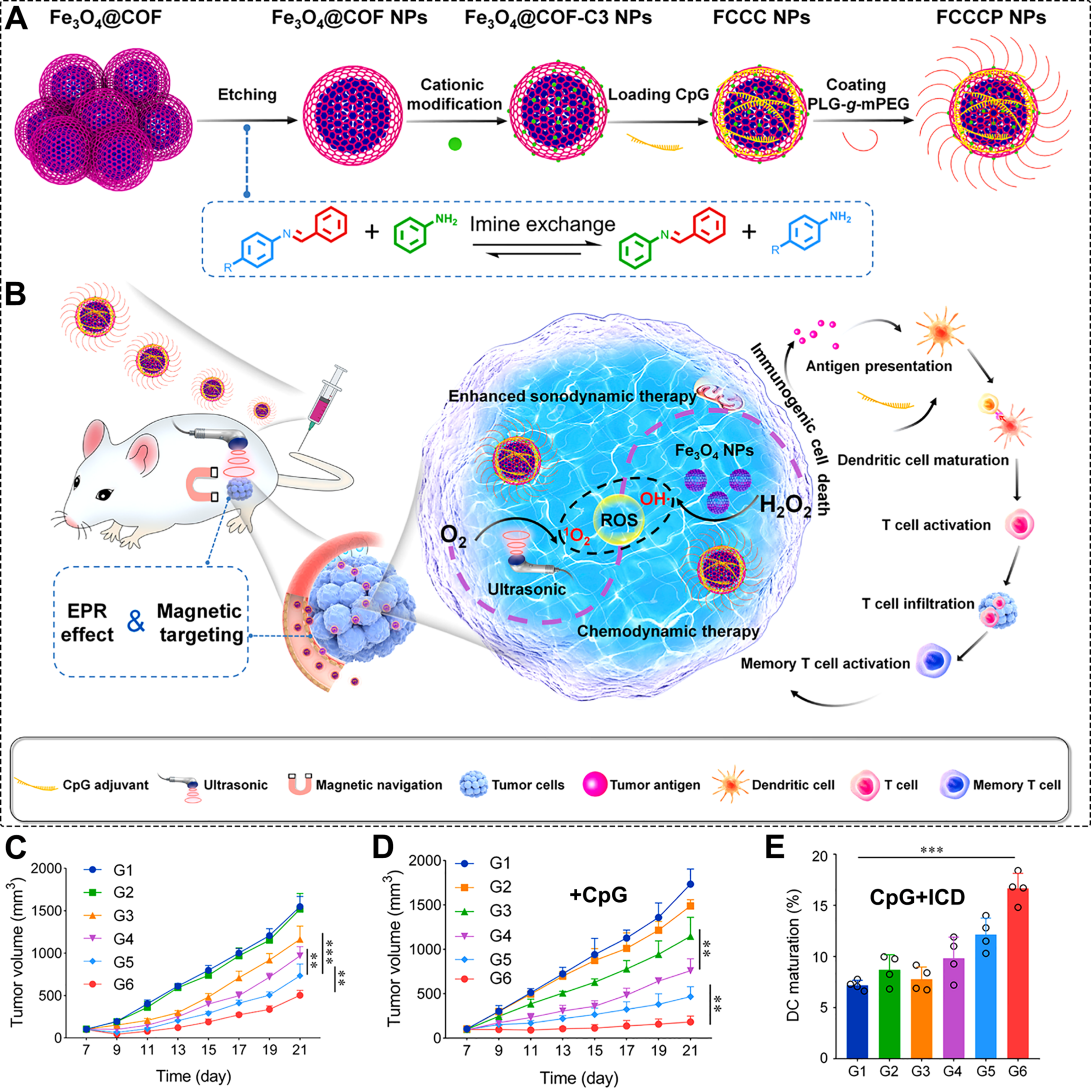
**(A)** Synthetic procedure of FCCCP NPs. **(B)** Schematic illustration of FCCCP NPs for combined SDT and CDT with immunotherapy against cancer cells. **(C)** Tumor volume changes during inhibition of cancer cell proliferation by FCCCP NPs compared with multiple experimental groups. **(D)** Mouse tumor volume changes following treatment with FCCCP NPs in combination with CpG. **(E)** Tumor volume changes when FCCCP NPs, CpG, and ICD are used together for combined cancer therapy. Reproduced with permission from Ref (196). Copyright (2024), Elsevier. **p <0.01,***p <0.001.

Regardless of the energy source, treatment efficacy heavily depends on the concentration of the sensitizer at the tumor site. Therefore, enhancing the targeting ability of COFs to diseased tissues becomes the next critical step. Wang et al. addressed this by developing core-shell Fe_3_O_4_@COF nanoparticles loaded with CpG adjuvant, yielding multifunctional FCCCP NPs (**Figure 8A**) (196). The Fe_3_O_4_ core provides CDT functionality and superparamagnetic properties, enabling active tumor targeting and potential deep-tumor SDT (**Figure 8B**). As shown in **Figure 8C**, FCCCP NPs effectively suppressed tumor growth in mice via SDT. When combined with CpG, the system achieved stronger tumor inhibition by coupling SDT with immune activation (**Figure 8D**). Flow cytometry analysis further revealed that both CpG and SDT increased the proportion of mature dendritic cells (DCs) in tumors, with their combination providing a solid foundation for enhanced immunotherapy (**Figure 8E**). This targeted COF platform not only delivers potent SDT but also induces ICD, releases tumor antigens, and synergizes with CpG, offering valuable insights for future nanotherapeutic design.

**Figure 9:**
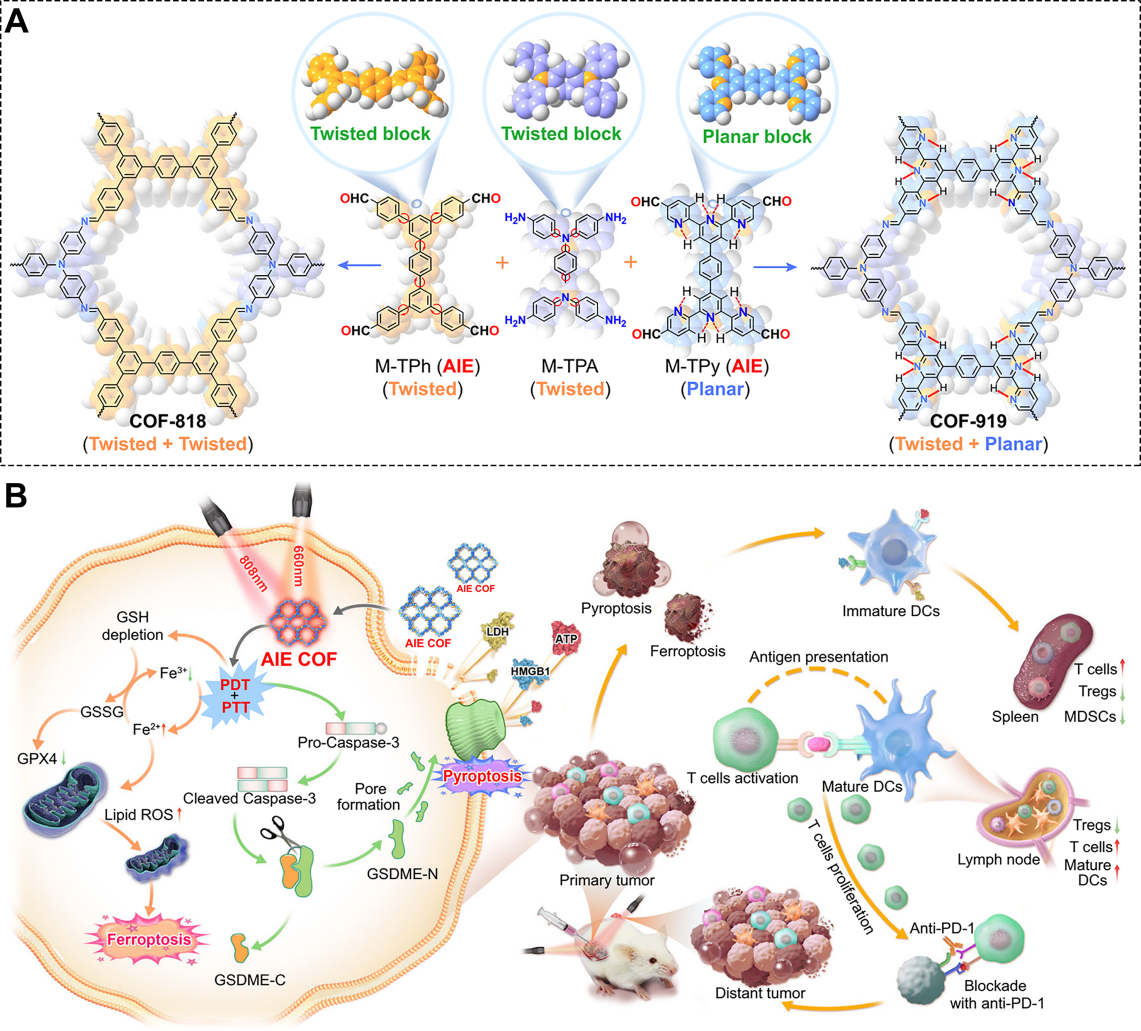
**(A)** Synthetic procedure and structure of COF-919. **(B)** Schematic illustration of COF-919 for combined PDT and PTT with pyroptosis-inducing and immunotherapeutic effects against tumors. Reproduced with permission from Ref (141). Copyright (2023), Springer Nature.

Following advancements in penetration depth and targeting efficiency, the research frontier is now shifting toward exploring different modes of cell death, aiming to go beyond classical ICD and induce more intense, highly immunogenic forms of death to fully activate the immune system. For instance, Sun et al. designed COF-919 using a tetrakis-aldehyde monomer (M-TPy, with AIE properties) and a triamine monomer (M-TPA) (**Figure 9A**) (141). Its unique planar-twisted structure enables strong near-infrared absorption, supporting excellent PDT and PTT performance (**Figure 9B**). This potent phototherapy triggers acute inflammation, activating Caspase-1 and forming large GSDMD-N pores, characteristic of pyroptosis (**Figure 9B**). Additionally, excessive ROS lead to lipid peroxidation, GSH depletion, and low GPX4 expression, inducing ferroptosis (**Figure 9B**). As highly immunogenic forms of programmed cell death, both pyroptosis and ferroptosis enhance antitumor immunity, significantly boosting the immunotherapeutic potential of this AIE-based COF. By synergistically combining these two mechanisms, the system elevates the ability of COFs to induce immunogenic cell death to a new level, achieving an effect greater than the sum of its parts.

In summary, through their customizable design, COF platforms not only overcome key limitations of conventional sensitizers-such as insufficient ROS generation, limited tissue penetration, and poor targeting-but also advance therapeutic paradigms from simple cell ablation to precisely inducing layered, immunogenic forms of cell death. This evolution provides a powerful and diverse arsenal for activating systemic antitumor immunity.

## 7 COF-mediated drug delivery and synergistic immunoadjuvant strategies

Powerful COF-based therapies successfully induce ICD, releasing a large number of tumor-associated antigens and danger signals-effectively providing the immune system with an accurate “map of the enemy.” However, to trigger a strong and lasting systemic immune attack, this “map” alone is insufficient. What is also needed are robust “supporting forces” capable of breaking immune tolerance, amplifying co-stimulatory signals, and empowering immune cells. Therefore, the role of COFs has evolved beyond that of a standalone therapeutic agent into a highly intelligent platform for immune drug delivery. By enabling spatiotemporally controlled delivery, COFs can precisely transport immunomodulators to the core of the tumor battlefield, transforming initial immune signals into powerful effector responses.

**Figure 10:**
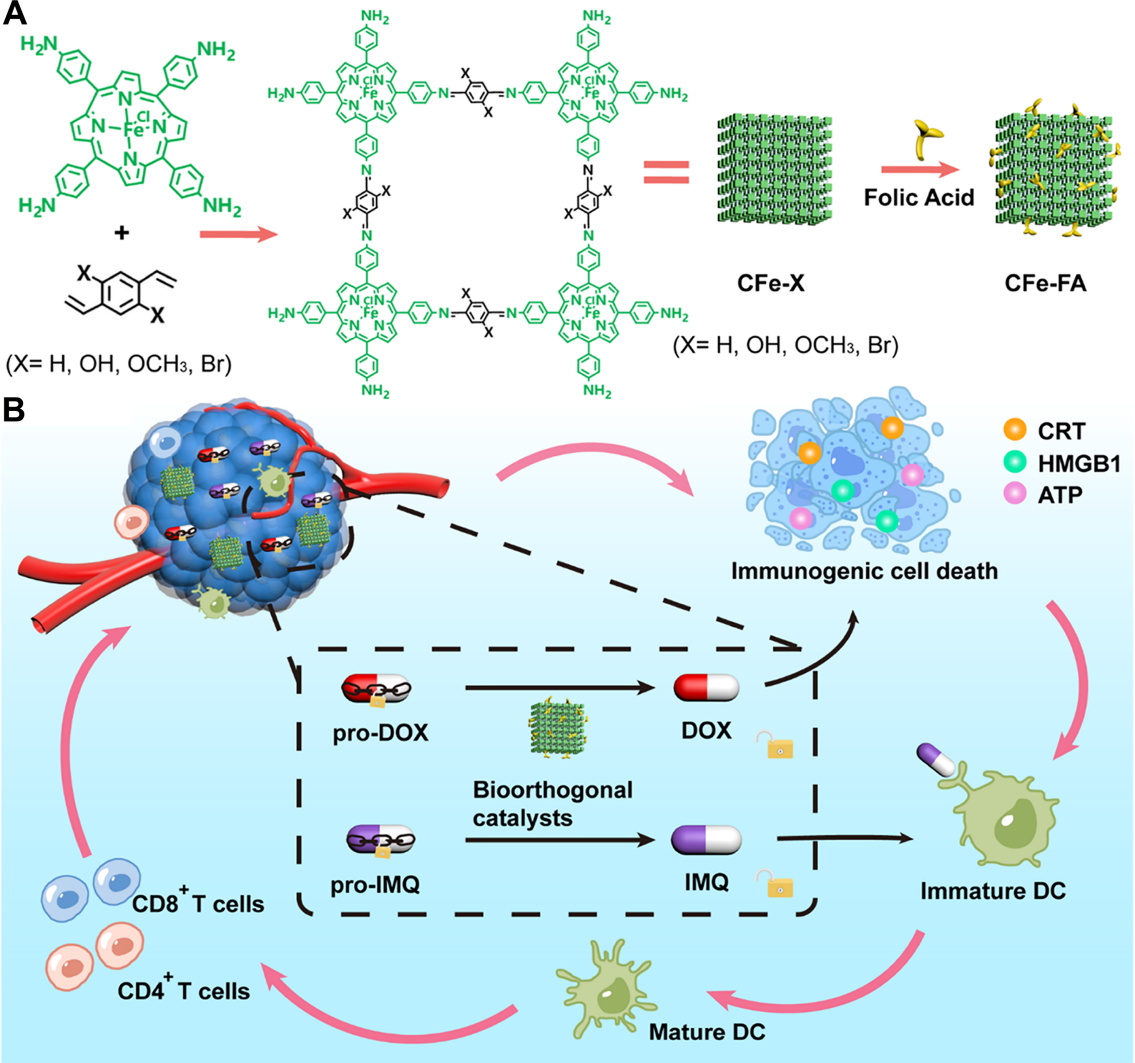
**(A)** Synthetic procedure of the CFe-FA composite. **(B)** Schematic illustration of CFe-FA composite enabling bioorthogonal activation of an *in situ* vaccine for highly effective cancer immunotherapy. Reproduced with permission from Ref (169). Copyright (2023), American Chemical Society.

The inherent high porosity and functionalizable surface of COFs make them ideal carriers for various immunomodulatory drugs, allowing for strong synergies with multiple therapeutic modalities. Beyond simple drug delivery, researchers have begun exploring the use of a carrier’s intrinsic bioorthogonal catalytic activity to locally activate prodrugs within the tumor. While studies on using COFs for bioorthogonal catalysis to activate prodrugs *in situ* are still limited, this approach holds significant promise. For example, Qu et al. developed a bioorthogonal activation platform based on porphyrin COFs to create an *in-situ* cancer vaccine (169). Specifically, they synthesized a ferrous iron (Fe^2+^)-coordinated porphyrin-based COF catalyst (CFe-X) (**Figure 10A**), which was further modified with folic acid to yield the multifunctional CFe-FA (**Figure 10A**). The folic acid modification enhanced tumor accumulation of the nanosystem. The authors found that this COF-based Fe^2+^ catalyst provided abundant active sites to locally activate a doxorubicin prodrug, while simultaneously releasing tumor-associated antigens (TAAs) and triggering ICD (**Figure 10B**). Additionally, the catalyst could activate the TLR7/8 agonist prodrug pro-imiquimod (pro-IMQ), further amplifying immune stimulation (**Figure 10B**). This system offers a novel paradigm for constructing personalized *in situ* vaccines through bioorthogonal chemistry. More importantly, by simultaneously activating both a chemotherapeutic prodrug and an immunoadjuvant prodrug, the strategy maximizes therapeutic efficacy while nearly eliminating systemic side effects-achieving safe and efficient “*in situ* vaccination.” Porphyrin-based COFs represent excellent nanoplatforms for PDT and PTT. Both PDT and PTT can activate immunotherapy against murine cancer cells by inducing ICD; however, their efficacy is typically constrained by the limited tissue penetration depth of light. In contrast, although X-ray-activated radiotherapy-radiodynamic therapy (RT-RDT) can also trigger antitumor immune responses, it poses safety concerns for normal tissues due to high radiation doses. In this work, Qu et al. developed this COF system loaded with a chemotherapeutic prodrug and ingeniously leveraged the catalytic activity of Fe^2+^ ions coordinated within the porphyrin units of the COF structure to achieve highly efficient *in situ* immunotherapy. This study not only represents a pioneering advancement of COFs in the field of biocatalysis but also offers a novel strategy for the development of cancer immunotherapeutic vaccines.

**Figure 11:**
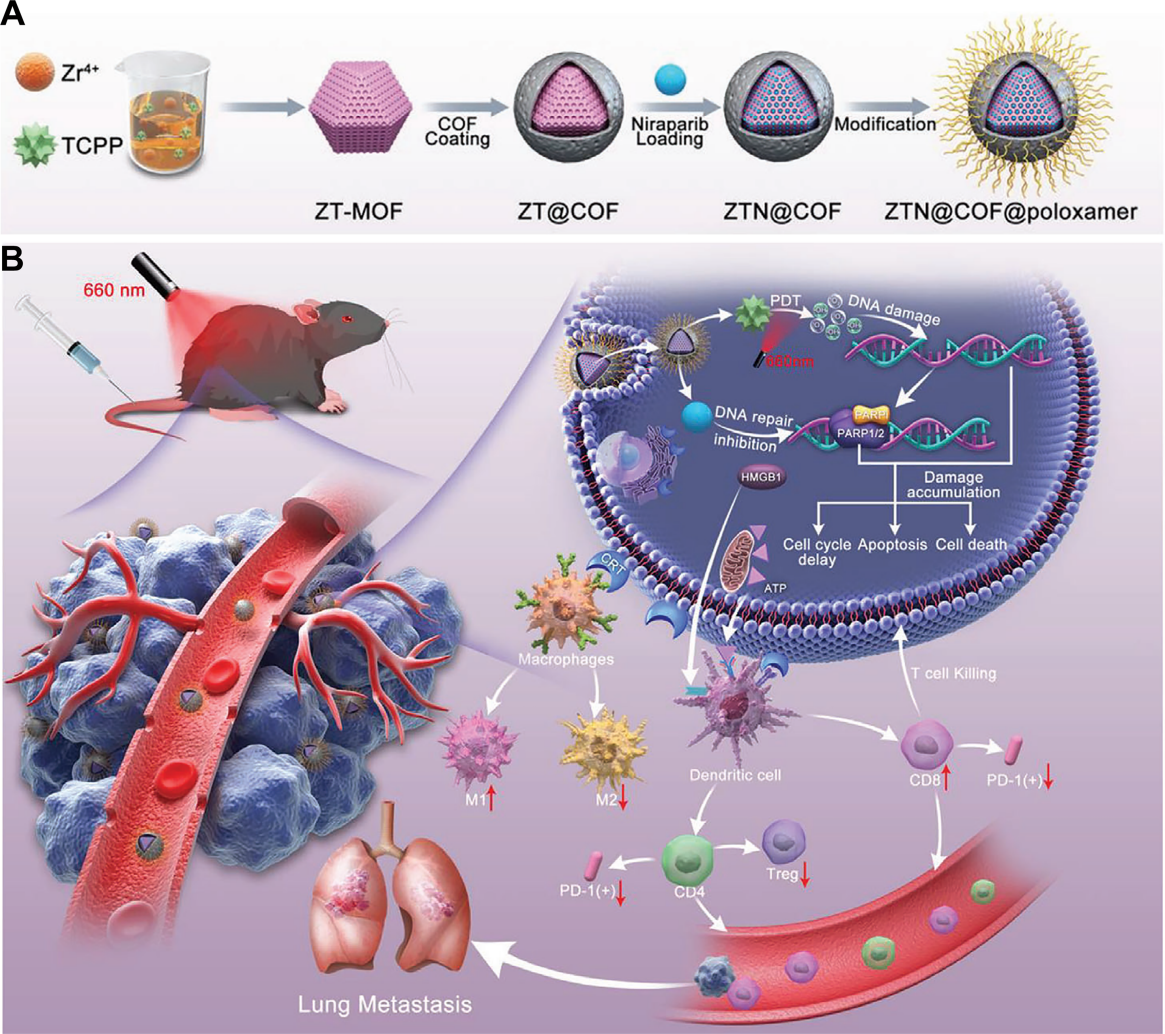
**(A)** Synthetic procedure of the ZTN@COF@poloxamer composite. **(B)** Schematic illustration of tumor suppression by ZTN@COF@poloxamer through PDT combined with PARP inhibition and activation of antitumor immune responses. Reproduced with permission from Ref (197). Copyright (2024), Wiley-VCH GmbH.

For established therapeutic agents, COFs can enhance drug efficacy and overcome resistance through precise delivery. It is well known that cancer cells can develop resistance to ROS-induced DNA damage via repair mechanisms. To counter this, combining therapy with PARP inhibitors presents a promising solution. Wen et al. addressed this by fabricating a MOF@COF core-shell nanocapsule, termed ZTN@COF@poloxamer (**Figure 11A**) (197). Upon light irradiation, the porphyrin component in the composite enables PDT. Meanwhile, the MOF@COF structure effectively loads and releases niraparib, a PARP inhibitor, which suppresses DNA repair and significantly inhibits the growth of soft tissue sarcomas (**Figure 11B**). In this system, the MOF@COF nanocapsule utilizes π-π stacking interactions to efficiently load the hydrophobic drug niraparib and ensure its controlled release. The authors demonstrated that the PDT effect induces ICD, triggering antigen release and immune activation (**Figure 11B**), and that niraparib itself can also promote ICD. The combined immune effects from both PDT and niraparib not only suppress the growth of distant tumors in mice but also prevent lung metastasis. This work further highlights the potential of COF hybrid materials in treating diverse cancers, including sarcomas. In addition, this research has uncovered a novel phenomenon: encapsulating a non-photosensitive COF shell around a porphyrin-based MOF photosensitizer (Zr-TCPP) can surprisingly enhance its capacity to generate ROS. This amplified ROS production offers greater potential for inducing robust ICD. Although the Zr-TCPP MOF itself is capable of loading therapeutic agents such as niraparib, the formation of a MOF@COF core-shell architecture significantly improves drug-loading performance. The COF shell not only provides additional porous channels but also strengthens drug binding through π-π stacking interactions within its covalent framework. As a result, the overall drug-loading capacity of the nanoplatform is greatly enhanced. Notably, in this system, the COF serves not merely as a high-efficiency drug carrier; it also contributes to superior anticancer efficacy by forming a synergistic porous composite with the MOF. Thus, this strategic design presents a promising therapeutic approach, even against highly aggressive soft tissue sarcomas. Although this PDT/PARPi combination demonstrates impressive synergistic antitumor and immunostimulatory effects, a more systematic evaluation of its therapeutic window, optimal light dosing, and inclusion of rigorous control groups is needed to fully substantiate its clinical translatability.

**Figure 12:**
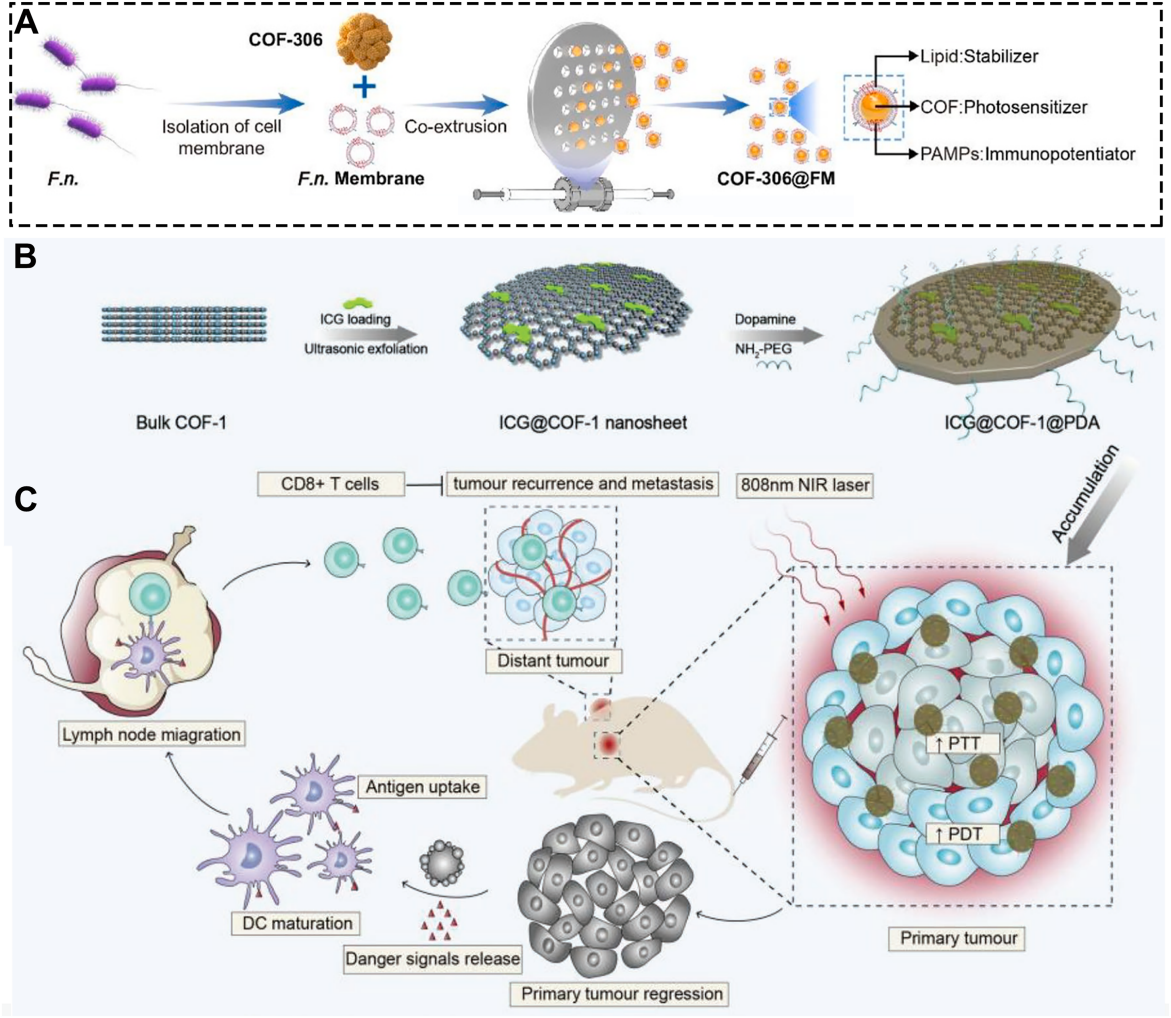
**(A)** Synthetic procedure of the COF-306@FM. Reproduced with permission from Ref (170). Copyright (2025), KeAi Communications Co. **(B)** Synthetic procedure of the ICG@COF-1@PDA material. **(C)** Schematic illustration of ICG@COF-1@PDA for combined PDT, PTT, and immunotherapy against tumors. Reproduced with permission from Ref (158). Copyright (2019), Wiley-VCH GmbH.

Effective drug delivery first requires that the carrier be efficiently internalized by cells and uniformly distributed deep within the tumor tissue. Thus, enhancing the stability of COF nanoplatforms in circulation and their cellular uptake is critical. To this end, Sun et al. wrapped COF materials with *Fusobacterium nucleatum* (F.n.), a bacterium commonly found in tumors, to create COF-306@FM (**Figure 12A**) (170). The bacterial membrane coating enables efficient cellular uptake and promotes uniform distribution throughout the tumor. Conventional phototherapeutic COFs often suffer from low cellular internalization, limiting their efficacy. In this system, the bacterial membrane not only prevents COF aggregation but also enhances the PDT-induced immune response. Overall, COF-306@FM achieves high tumor cell uptake and deep tissue penetration. Moreover, the bacterial membrane itself acts as a potent immunoadjuvant, effectively “heating up” cold tumors and significantly improving response rates to immune checkpoint inhibitors. This strategy addresses a core challenge in COF-based drug delivery: low delivery efficiency.

COFs have already demonstrated significant potential in biomedicine. Even conventional drugs can be significantly enhanced when loaded onto COFs. For instance, Yuan et al. loaded the photothermal agent ICG onto COF nanosheets (**Figure 12B**) (158). By dispersing ICG molecules at the single-molecule level within the COF structure, this system effectively prevents ICG aggregation and quenching, thereby greatly improving its phototherapeutic performance. As a result, it elicits a robust antitumor immune response and suppresses distant metastasis (**Figure 12C**). This study highlights the broad utility of COFs as a general platform for enhancing the performance of conventional therapeutic agents.

## 8 Multimodal imaging-guided and integrated immunotherapy

Following the successful use of COFs for therapeutic intervention, microenvironment modulation, and drug delivery, a critical next challenge arises: how to visualize and evaluate the ongoing immune “battle” within the tumor in real time. The realization of precision medicine hinges on the ability to monitor treatment processes dynamically and assess therapeutic outcomes accurately. The highly tunable framework of COFs allows for the easy integration of components required for various imaging modalities, enabling a seamless fusion of therapeutic and diagnostic functions. This theranostic design not only facilitates precise tumor localization and treatment guidance but also enables real-time visualization of dynamic changes in the tumor microenvironment (TME), including ROS levels, GSH depletion, and O_2_ generation. These capabilities provide an essential informational window for optimizing therapeutic strategies.

**Figure 13:**
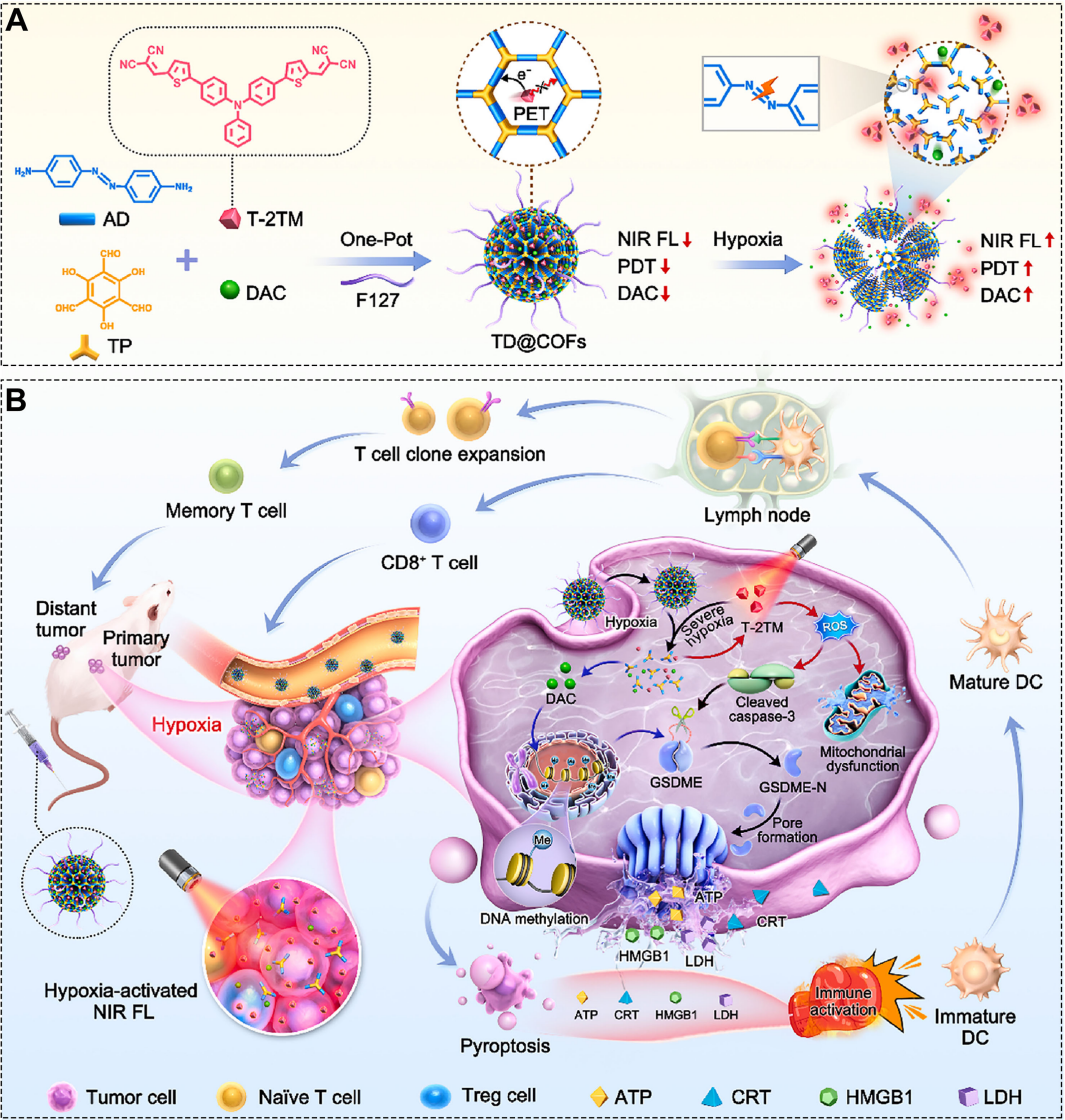
**(A)** Synthetic procedure of the TD@COFs composite. **(B)** Schematic illustration of TD@COFs for combined tumor suppression through PDT, hypoxia-activated pyroptosis, and the resulting immune response. Reproduced with permission from Ref (157). Copyright (2024), Elsevier.

For instance, Liu et al. developed a composite material by combining COFs with MnO_2_. The MnO_2_ shell consumes GSH while releasing Mn^2+^ ions, which generate strong magnetic resonance imaging (MRI) signals. This allows real-time monitoring of TME reversal, offering dynamic guidance for therapy. Beyond tracking therapy-induced environmental changes, a more advanced strategy involves designing imaging signals that are highly specific to the TME, enabling “activatable” precision diagnosis. Building on this concept, Bing et al. developed TD@COFs-a multifunctional material that simultaneously carries a pyroptosis-inducing drug-using a one-pot synthesis approach (**Figure 13A**) (157). Due to intramolecular photoinduced electron transfer (PET), the COF exhibits weak fluorescence and limited photodynamic activity. However, under the hypoxic conditions of tumor cells, the azo bonds in the COF structure are activated, triggering degradation and drug release. This process simultaneously enhances the fluorescence of the AIE-active component and significantly boosts the material’s PDT efficiency (**Figure 13A**). The resulting pyroptosis-mediated immune response effectively suppresses the growth of distant 4T1 tumors (**Figure 13B**). Notably, the COF nanoparticles remain “off” in normal tissues, with both fluorescence and PDT effects quenched, but are selectively “switched on” in the hypoxic tumor environment, producing strong fluorescent signals and ROS generation (**Figure 13B**). This “on-off” design enables ultra-high signal-to-noise-ratio imaging, significantly improving the accuracy of image guidance and enhancing treatment safety. It is worth noting that, despite the promising theranostic integration demonstrated above, modality-specific challenges remain. For instance, the quantitative relationship between Mn^2+^ release and MRI relaxivity requires further optimization to enhance imaging sensitivity. Moreover, although PET-based fluorescence switching enables effective “off-on” activation, the extent of initial quenching and the magnitude of signal recovery upon activation are highly dependent on the COF’s electronic structure and local microenvironment, necessitating precise molecular engineering to balance diagnostic signal-to-noise ratio with therapeutic output.

## 9 Exploring novel immune activation mechanisms and long-term effects

Theranostic COF platforms provide powerful tools for probing the tumor immune microenvironment, and the insights gained are now driving researchers toward more advanced paradigms of immune activation-such as combining boron neutron capture therapy (BNCT) with immunotherapy. Moving beyond the traditional concept of ICD, current research aims to leverage the precise biological effects of COFs to manipulate fundamental immune processes. This includes the *in situ* formation of tertiary lymphoid structures (TLS) within tumors-resembling lymphoid organs-to establish long-term immune fortresses, as well as investigating extraordinary phenomena like abscopal effects triggered by localized nuclear reactions. These advances mark a shift in COF research from simple tool application to active immune programming of biological systems.

**Figure 14:**
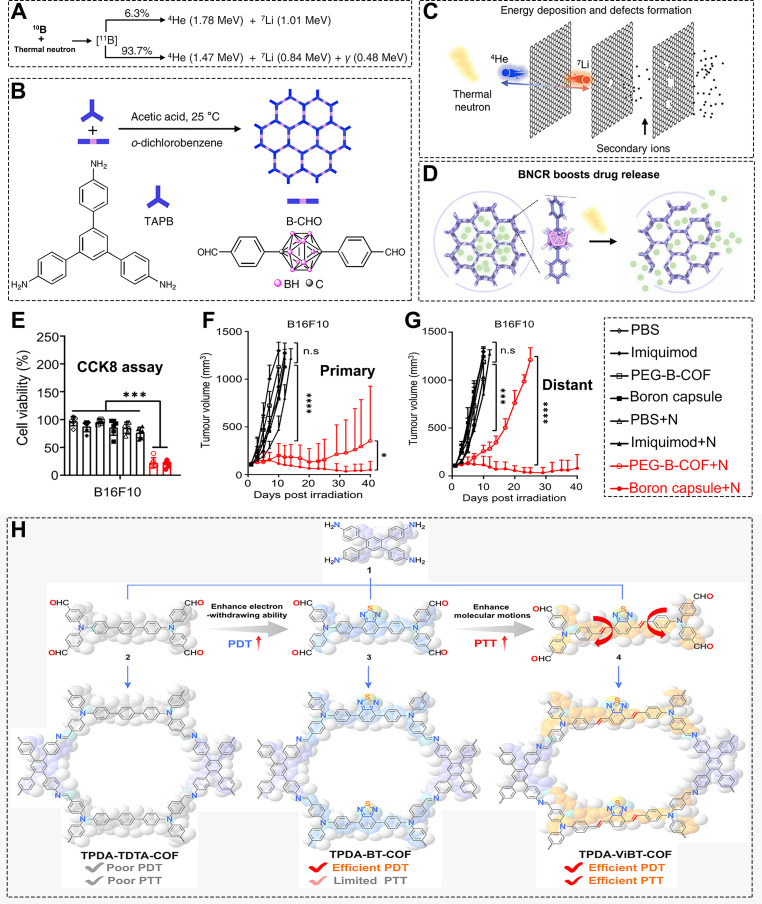
**(A)** Antitumor mechanism of boron neutron capture therapy (BNCT). **(B)** Synthetic procedure of B-COF. **(C)** Mechanism of drug release from B-COF upon thermal neutron irradiation. **(D)** Schematic illustration of BNCR-triggered drug release. **(E)** Cell viability of B16F10 cancer cells measured by CCK-8 assay. **(F, G)** Tumor growth inhibition of bilateral tumors by PEG-B-COF and different experimental groups. Reproduced with permission from Ref (198). Copyright (2023), Springer Nature. **(H)** Synthetic procedures and structures of three different COF materials (TPDA-TDTA-COF, TPDA-BT-COF, and TPDA-ViBT-COF). Reproduced with permission from Ref (168). Copyright (2025), Springer Nature. ***p <0.001,****p <0.0001.

For example, Liu et al. conducted a groundbreaking study that extended COF applications into the emerging field of boron neutron capture therapy (BNCT), which has attracted significant attention since its clinical approval in 2020 (198). BNCT is a binary radiotherapy approach in which neutron irradiation activates ^10^B atoms, triggering the release of high-energy particles (^4^He and ^7^Li) within cancer cells, leading to their destruction (**Figure 14A**) (198). The authors first synthesized a carborane-based COF (B-COF) (**Figure 14B**), which was then coated with DSPE-PEG to improve dispersion stability, yielding PEG-B-COF. Upon neutron irradiation, the COF not only released high-energy He and Li particles (**Figure 14C**) but also gradually released loaded imiquimod due to structural defects in the COF capsule (**Figure 14D**), promoting macrophage polarization and enhancing immune responses. The CCK-8 assay serves as an effective method for evaluating the therapeutic efficacy of nanomedicines (211–214). CCK-8 assays in this work showed that both PEG-B-COF and the boron capsule effectively inhibited B16F10 cell proliferation under neutron irradiation (**Figure 14E**). *In vivo* experiments (**Figure 14F**) further confirmed that mice treated with the boron capsule plus neutron irradiation exhibited the most significant tumor suppression. In abscopal effect studies (**Figure 14G**), the same treatment also showed the strongest inhibition of distant B16F10 tumors. While PEG-COF with neutron irradiation showed some abscopal effect, its immune stimulation was limited. This work not only demonstrated the high cytotoxic efficiency of neutron-activated nuclear reactions but also, for the first time, reported the abscopal effect induced by BNCT. By revealing the detailed immune activation mechanisms through single-cell sequencing, the study achieved simultaneous regression of primary and distant tumors, establishing a novel paradigm for radio-immunotherapy. Although the pioneering BNCT-COF study by Liu et al. demonstrated promising antitumor effects, its translational potential warrants more rigorous scrutiny, particularly regarding whether the applied neutron dose falls within a clinically safe therapeutic window, whether the tumor-to-normal tissue ^10^B accumulation ratio is sufficient for selective cytotoxicity, and whether appropriate control groups were included to rule out nonspecific effects. Future studies of this nature should systematically report radiation parameters, pharmacokinetic profiles, and dose-dependent immune activation to enhance reproducibility, comparability, and clinical relevance.

The realization of abscopal effects relies on robust systemic immunity, and the formation of tertiary lymphoid structures (TLS) is key to establishing long-lasting, high-efficiency antitumor immunity. Sun et al. discovered a surprising new function of COF-mediated phototherapy: the induction of TLS formation (168). In this study, they designed and synthesized three highly luminescent AIE-type COFs (TPDA-TDTA-COF, TPDA-BT-COF, and TPDA-ViBT-COF) (**Figure 14H**). Photophysical characterization revealed that TPDA-ViBT-COF exhibited the strongest PDT and PTT capabilities due to its unique electronic structure. Subsequent *in vitro* and *in vivo* experiments demonstrated that the excellent phototherapeutic performance of TPDA-ViBT-COF effectively reversed the immunosuppressive TME and stimulated the host defense system. The resulting strong inflammatory response promoted the formation of inducible TLS (iTLS) and significantly enhanced immunotherapy. The study showed that COFs promoted cytokine secretion, driving the maturation and recruitment of T and B cells, thereby forming lymph node-like immune hubs within the tumor. Moreover, as previously mentioned, another study by Sun et al. demonstrated that bacterial membrane-camouflaged COF (COF-306@FM) also effectively induced TLS formation by enhancing the infiltration of CD8^+^ T cells and B cells (170). These findings strongly suggest that COF platforms-whether in their native form or through bioinspired modifications-possess unique and broadly applicable advantages in reshaping the tumor immune architecture and eliciting the most effective antitumor immune responses, laying a solid theoretical foundation for their clinical translation.

## 10 Conclusion

This review systematically highlights COFs as an emerging nanoplatform, showcasing their exceptional design flexibility and remarkable therapeutic potential in cancer immunotherapy. Through precise engineering of their modular structures, COFs have successfully integrated multiple critical functions into a single platform. First, they can act as highly efficient sensitizers, significantly enhancing photodynamic, sonodynamic, radio-, and chemodynamic therapies. By triggering robust ICD, COFs release tumor antigens and danger signals, effectively delivering the “first signal” required to initiate anti-tumor immune responses. Second, the inherent high porosity and easily modifiable surface of COFs make them ideal carriers for immunoadjuvants (such as CpG or Poly(I:C)), checkpoint inhibitors, or prodrugs. This enables the coordinated accumulation and controlled release of therapeutic agents within the tumor site, providing a well-stocked “arsenal” for systemic immune activation. More importantly, many COF materials exhibit intrinsic enzyme-like catalytic activities-such as catalase- or glutathione peroxidase-mimicking properties, that actively reprogram the immunosuppressive TME. These materials can convert endogenous H_2_O_2_ into O_2_ to alleviate hypoxia, while simultaneously depleting overexpressed GSH to reduce ROS scavenging. In doing so, they fundamentally transform immune-quiescent “cold” tumors into inflamed, immune-responsive “hot” tumors. Furthermore, cutting-edge research into COFs’ ability to induce novel inflammatory cell death pathways, including pyroptosis, ferroptosis, and PANoptosis, as well as to promote the formation of tertiary lymphoid structures (TLS), has opened new avenues for generating strong and long-lasting anti-tumor immune memory. Taken together, the structural tunability, functional integrability, and responsive controllability of COFs perfectly meet the urgent need for a unified strategy in cancer immunotherapy-one that simultaneously modulates the microenvironment, achieves potent tumor killing, and activates immunity. This progress marks a pivotal shift from the era of simple drug delivery toward a new frontier of intelligent “immune engineering.”

Despite the highly promising results of COF-based immunotherapies in preclinical studies, their translation into clinical practice and widespread application faces several critical challenges that must be urgently addressed. First, the long-term stability of COFs under physiological conditions remains insufficiently understood. Certain COFs linked via imine or boronate ester bonds may undergo structural degradation in acidic lysosomal environments or under oxidative stress, which could compromise both their functional performance and biocompatibility. However, for certain COF-based carriers, structural degradation and disassembly can facilitate the release of the loaded therapeutic agents. Second, while COFs are generally considered biocompatible, their use as carriers for photosensitizers in PDT carries a risk of nonspecific phototoxicity. This concern becomes particularly significant when light dosing is poorly controlled or when healthy tissues are unintentionally exposed to irradiation. Third, certain porphyrin-based photosensitizers may be susceptible to photobleaching, which can compromise the phototherapeutic efficacy of porphyrin-containing COF nanoplatforms. Fourth, the clinical translation of COF-based nanoplatforms is hindered by several critical challenges, including potential immunogenicity, off-target accumulation in healthy tissues, and an incomplete understanding of their long-term metabolic fate and clearance pathways. Future efforts must therefore balance therapeutic innovation with rigorous assessment of biocompatibility, biodistribution, and pharmacokinetics to bridge the gap between laboratory breakthroughs and clinical reality. Fifth, large-scale synthesis and quality control present practical bottlenecks. Most current COF synthesis methods fall short of meeting Good Manufacturing Practice (GMP) standards in terms of yield, batch-to-batch consistency, cost efficiency, and the ability to produce sterile, pyrogen-free materials. Finally, there is still room for improvement in delivery efficiency *in vivo*. Overcoming complex biological barriers, enhancing tumor-targeted accumulation, and achieving deep tissue penetration remain key areas requiring continuous optimization.

To address these challenges, future research may pursue breakthroughs through multiple strategies. First, material design should prioritize biocompatible and biodegradable building blocks-such as amino acids or nucleotide derivatives-and incorporate stimulus-responsive linkages (e.g., sensitive to pH, GSH, or enzymes) to enable controlled degradation and safe clearance after therapy. Second, green, efficient, and scalable synthesis techniques, such as microwave-assisted reactions or continuous flow chemistry-should be developed to improve production efficiency while ensuring product uniformity and stability. Third, the design of non-porphyrin photosensitizing units should be actively pursued, with a focus on systematically evaluating the phototherapeutic performance of a broader range of photoactive molecules. Fourth, advanced biomimetic functionalization approaches, such as cell membrane coating (using red blood cell, cancer cell, or bacterial membranes), could be employed to endow COF nanoparticles with prolonged blood circulation, enhanced active targeting, and immune evasion capabilities, thereby optimizing their pharmacokinetic profiles. Finally, fostering deep interdisciplinary collaboration is essential. Close cooperation among material scientists, pharmacologists, immunologists, and clinicians is needed-not only to assess therapeutic efficacy but also to design rational preclinical and clinical studies grounded in real clinical needs. Such collaboration will accelerate the transformation of groundbreaking laboratory discoveries into tangible therapies that benefit patients. To better delineate a forward-looking research agenda, we propose three actionable priorities: (1) establishing quantitative models that correlate the *in vivo* degradation kinetics of COFs with the magnitude of immune activation they elicit; (2) developing synthesis protocols and quality control standards compliant with Good Manufacturing Practice (GMP) for clinical translation; and (3) engineering intelligent COF-based probes capable of real-time monitoring of dynamic changes in the tumor microenvironment. These specific objectives are expected to accelerate the field’s progression from proof-of-concept studies toward clinical application. Although the path ahead is long, the immense potential of COFs firmly lays the foundation for their eventual success in the field of precision immunotherapy.

## References

[1] Deo SVS Sharma J Kumar S . GLOBOCAN 2020 report on global cancer burden: challenges and opportunities for surgical oncologists. Ann Surg Oncol. (2022) 29:6497–500. doi: 10.1245/s10434-022-12151-6, PMID: 35838905

[2] Trapani D Ginsburg O Fadelu T Lin NU Hassett M Ilbawi AM . Global challenges and policy solutions in breast cancer control. Cancer Treat Rev. (2022) 104:102339. doi: 10.1016/j.ctrv.2022.102339, PMID: 35074727

[3] van Hoogstraten LMC Vrieling A van der Heijden AG Kogevinas M Richters A Kiemeney LA . Global trends in the epidemiology of bladder cancer: challenges for public health and clinical practice. Nat Rev Clin Oncol. (2023) 20:287–304. doi: 10.1038/s41571-023-00744-3, PMID: 36914746

[4] Pointer KB Pitroda SP Weichselbaum RR . Radiotherapy and immunotherapy: open questions and future strategies. Trends Cancer. (2022) 8:9–20. doi: 10.1016/j.trecan.2021.10.003, PMID: 34740553

[5] Zhang Z Liu X Chen D Yu J . Radiotherapy combined with immunotherapy: the dawn of cancer treatment. Signal Transduction Targeted Ther. (2022) 7:258. doi: 10.1038/s41392-022-01102-y, PMID: 35906199 PMC9338328

[6] Kamrani A Hosseinzadeh R Shomali N Heris JA Shahabi P Mohammadinasab R . New immunotherapeutic approaches for cancer treatment. Pathol. Res Pract. (2023) 248:154632. 37480597 10.1016/j.prp.2023.154632

[7] Kaur R Bhardwaj A Gupta S . Cancer treatment therapies: traditional to modern approaches to combat cancers. Mol Biol Rep. (2023) 50:9663–76. doi: 10.1007/s11033-023-08809-3, PMID: 37828275

[8] Topalian SL Forde PM Emens LA Yarchoan M Smith KN Pardoll DM . Neoadjuvant immune checkpoint blockade: A window of opportunity to advance cancer immunotherapy. Cancer Cell. (2023) 41:1551–66. doi: 10.1016/j.ccell.2023.07.011, PMID: 37595586 PMC10548441

[9] Stoop TF Theijse RT Seelen LWF Groot Koerkamp B van Eijck CHJ Wolfgang CL . C. International Collaborative Group on Locally Advanced Pancreatic, Preoperative chemotherapy, radiotherapy and surgical decision-making in patients with borderline resectable and locally advanced pancreatic cancer. Nat Rev Gastroenterol Hepatol. (2024) 21:101–24. doi: 10.1038/s41575-023-00856-2, PMID: 38036745

[10] Dagher OK Schwab RD Brookens SK Posey AD Jr . Advances in cancer immunotherapies. Cell. (2023) 186:1814–1814.e1. doi: 10.1016/j.cell.2023.02.039, PMID: 37059073

[11] Oliveira G Wu CJ . Dynamics and specificities of T cells in cancer immunotherapy. Nat Rev Cancer. (2023) 23:295–316. doi: 10.1038/s41568-023-00560-y, PMID: 37046001 PMC10773171

[12] Dou Y Wang Y Tian S Song Q Deng Y Zhang Z . Metal–organic framework (MOF)-based materials for pyroptosis-mediated cancer therapy. Chem Commun. (2024) 60:6476–87. doi: 10.1039/D4CC02084G, PMID: 38853690

[13] Li Y Song Y Yin J Pan W Li N Tang B . Organelle-based immunotherapy strategies for fighting against cancer. Chem Commun. (2024) 60:8170–85. doi: 10.1039/D4CC01594K, PMID: 38979965

[14] Lu Q Kou D Lou S Ashrafizadeh M Aref AR Canadas I . Nanoparticles in tumor microenvironment remodeling and cancer immunotherapy. J Hematol Oncol. (2024) 17:16. doi: 10.1186/s13045-024-01535-8, PMID: 38566199 PMC10986145

[15] Ma K Chen Z Liang K Pei Y Pei Z . A near-infrared light-driven Janus nanomotor for deep tumor penetration and enhanced tumor immunotherapy. Chem Commun. (2024) 60:9550–3. doi: 10.1039/D4CC03445G, PMID: 39150078

[16] Xue L Thatte AS Mai D Haley RM Gong N Han X . Responsive biomaterials: optimizing control of cancer immunotherapy. Nat Rev Mater. (2024) 9:100–18. doi: 10.1038/s41578-023-00617-2

[17] Lahiri A Maji A Potdar PD Singh N Parikh P Bisht B . Lung cancer immunotherapy: progress, pitfalls, and promises. Mol Cancer. (2023) 22:40. doi: 10.1186/s12943-023-01740-y, PMID: 36810079 PMC9942077

[18] Sharma P Goswami S Raychaudhuri D Siddiqui BA Singh P Nagarajan A . Immune checkpoint therapy—current perspectives and future directions. Cell. (2023) 186:1652–69. doi: 10.1016/j.cell.2023.03.006, PMID: 37059068

[19] Xie N Shen G Gao W Huang Z Huang C Fu L . Neoantigens: promising targets for cancer therapy, *Signal Transduction Targeted Ther*. (2023) 8:9. doi: 10.1038/s41392-022-01270-x, PMID: 36604431 PMC9816309

[20] Hu J Arvejeh PM Bone S Hett E Marincola FM Roh K-H . Nanocarriers for cutting-edge cancer immunotherapies. J Transl Med. (2025) 23:447. doi: 10.1186/s12967-025-06435-0, PMID: 40234928 PMC12001629

[21] Postow MA Callahan MK Wolchok JD . Immune checkpoint blockade in cancer therapy. J Clin Oncol. (2015) 33:1974–82. doi: 10.1200/JCO.2014.59.4358, PMID: 25605845 PMC4980573

[22] Arafat Hossain M . A comprehensive review of immune checkpoint inhibitors for cancer treatment. Int Immunopharmacol. (2024) 143:113365. doi: 10.1016/j.intimp.2024.113365, PMID: 39447408

[23] Arya SP Thennakoon SKS Phuoc CMT Silwal AP Jahan R Postema RM . Aptamer-assisted phage display: enhancing checkpoint inhibition with a peptide and an aptamer targeting distinct sites on a single PD-L1 protein. Chem Commun. (2024) 60:7570–3. doi: 10.1039/D4CC02132K, PMID: 38940673

[24] Wei J Li W Zhang P Guo F Liu M . Current trends in sensitizing immune checkpoint inhibitors for cancer treatment. Mol Cancer. (2024) 23:279. doi: 10.1186/s12943-024-02179-5, PMID: 39725966 PMC11670468

[25] Zhao C Tang X Chen X Jiang Z . Multifaceted carbonized metal–organic frameworks synergize with immune checkpoint inhibitors for precision and augmented cuproptosis cancer therapy. ACS Nano. (2024) 18:17852–68. doi: 10.1021/acsnano.4c04022, PMID: 38939981

[26] Hwang WL Pike LRG Royce TJ Mahal BA Loeffler JS . Safety of combining radiotherapy with immune-checkpoint inhibition. Nat Rev Clin Oncol. (2018) 15:477–94. doi: 10.1038/s41571-018-0046-7, PMID: 29872177

[27] Howard FM Villamar D He G Pearson AT Nanda R . The emerging role of immune checkpoint inhibitors for the treatment of breast cancer. Expert Opin Invest. Drugs. (2022) 31:531–48., PMID: 34569400 10.1080/13543784.2022.1986002 PMC8995399

[28] Liu L Pan Y Zhao C Huang P Chen X Rao L . Boosting checkpoint immunotherapy with biomaterials. ACS Nano. (2023) 17:3225–58. doi: 10.1021/acsnano.2c11691, PMID: 36746639

[29] Goswami S Pauken KE Wang L Sharma P . Next-generation combination approaches for immune checkpoint therapy, Nat. Immunol. (2024) 25:2186–99., PMID: 39587347 10.1038/s41590-024-02015-4 PMC13112085

[30] Lynch C Pitroda SP Weichselbaum RR . Radiotherapy, immunity, and immune checkpoint inhibitors. Lancet Oncol. (2024) 25:e352–62. doi: 10.1016/S1470-2045(24)00075-5, PMID: 39089313

[31] Bashash D Zandi Z Kashani B Pourbagheri-Sigaroodi A Salari S Ghaffari SH . Resistance to immunotherapy in human Malignancies: Mechanisms, research progresses, challenges, and opportunities. J Cell Physiol. (2022) 237:346–72. doi: 10.1002/jcp.30575, PMID: 34498289

[32] Gupta S Shukla S . Limitations of immunotherapy in cancer. Cureus. (2022) p:e30856. doi: 10.7759/cureus.30856, PMID: 36465776 PMC9708058

[33] Kim J Lee BJ Moon S Lee H Lee J Kim B-S . Strategies to overcome hurdles in cancer immunotherapy. Biomater. Res. (2024) 28:0080. 39301248 10.34133/bmr.0080 PMC11411167

[34] Wang M Yu F Zhang Y . Present and future of cancer nano-immunotherapy: opportunities, obstacles and challenges. Mol Cancer. (2025) 24:26. doi: 10.1186/s12943-024-02214-5, PMID: 39827147 PMC11748575

[35] Lin C-Y Huang K-Y Kao S-H Lin M-S Lin C-C Yang S-C . Small-molecule PIK-93 modulates the tumor microenvironment to improve immune checkpoint blockade response. Sci Adv. (2023) 9:eade9944. doi: 10.1126/sciadv.ade9944, PMID: 37027467 PMC10081850

[36] Lin X Li F Guan J Wang X Yao C Zeng Y . Janus silica nanoparticle-based tumor microenvironment modulator for restoring tumor sensitivity to programmed cell death ligand 1 immune checkpoint blockade therapy. ACS Nano. (2023) 17:14494–507. doi: 10.1021/acsnano.3c01019, PMID: 37485850

[37] Fang T Cao X Wang L Chen M Deng Y Chen G . Bioresponsive and immunotherapeutic nanomaterials to remodel tumor microenvironment for enhanced immune checkpoint blockade. Bioact. Mater. (2024) 32:530–42., PMID: 38026439 10.1016/j.bioactmat.2023.10.023 PMC10660011

[38] Shen K-Y Zhu Y Xie S-Z Qin L-X . Immunosuppressive tumor microenvironment and immunotherapy of hepatocellular carcinoma: current status and prospectives. J Hematol Oncol. (2024) 17:25. doi: 10.1186/s13045-024-01549-2, PMID: 38679698 PMC11057182

[39] Yang M Pu L Yang S Chen Z Guo J Liu Y . Engineered antler stem cells derived exosomes potentiate anti-tumor efficacy of immune checkpoint inhibitor by reprogramming immunosuppressive tumor microenvironment. Chem Eng. J. (2024) 479:147421.

[40] Yao Z Qi C Zhang F Yao H Wang C Cao X . Hollow Cu2MoS4 nanoparticles loaded with immune checkpoint inhibitors reshape the tumor microenvironment to enhance immunotherapy for pancreatic cancer. Acta Biomater. (2024) 173:365–77. doi: 10.1016/j.actbio.2023.10.024, PMID: 37890815

[41] Luo Y Li C Zhang Y Liu P Chen H Zhao Z . Gradient tumor microenvironment-promoted penetrating micelles for hypoxia relief and immunosuppression reversion in pancreatic cancer treatment. Acta Biomater. (2023) 167:387–400., PMID: 37276955 10.1016/j.actbio.2023.05.047

[42] Yang J Zhang C Chen X Zhou D Sun Z Niu R . Ultra-efficient radio-immunotherapy for reprogramming the hypoxic and immunosuppressive tumor microenvironment with durable innate immune memory. Biomaterials. (2023) 302:122303. doi: 10.1016/j.biomaterials.2023.122303, PMID: 37689049

[43] Yang W Pan X Zhang P Yang X Guan H Dou H . Defeating melanoma through a nano-enabled revision of hypoxic and immunosuppressive tumor microenvironment. Int J Nanomed. (2023) 18:3711–25. doi: 10.2147/IJN.S414882, PMID: 37435153 PMC10332423

[44] He M Zhang M Xu T Xue S Li D Zhao Y . Enhancing photodynamic immunotherapy by reprograming the immunosuppressive tumor microenvironment with hypoxia relief. J Controlled Release. (2024) 368:233–50. doi: 10.1016/j.jconrel.2024.02.030, PMID: 38395154

[45] Li X Sun X Wang Y Chen H Gao Y . A nanotheranostics with hypoxia-switchable fluorescence and photothermal effect for hypoxia imaging-guided immunosuppressive tumor microenvironment modulation. J Colloid Interface Sci. (2025) 678:897–912. doi: 10.1016/j.jcis.2024.09.133, PMID: 39321645

[46] Liu X Li Y Wang K Chen Y Shi M Zhang X . GSH-responsive nanoprodrug to inhibit glycolysis and alleviate immunosuppression for cancer therapy. Nano Lett. (2021) 21:7862–9. doi: 10.1021/acs.nanolett.1c03089, PMID: 34494442

[47] Wang M Chang M Li C Chen Q Hou Z Xing B . Tumor-microenvironment-activated reactive oxygen species amplifier for enzymatic cascade cancer starvation/chemodynamic/immunotherapy. Adv Mater. (2022) 34:2106010. doi: 10.1002/adma.202106010, PMID: 34699627

[48] Wang J Zhang Q Li Y Pan X Shan Y Zhang J . Remodeling the tumor microenvironment by vascular normalization and GSH-depletion for augmenting tumor immunotherapy. Chin Chem Lett. (2024) 35:108746. doi: 10.1016/j.cclet.2023.108746

[49] Wang M Liu Y Li Y Lu T Wang M Cheng Z . Tumor microenvironment-responsive nanoparticles enhance IDO1 blockade immunotherapy by remodeling metabolic immunosuppression, adv. Sci. (2025) 12:2405845., PMID: 39661740 10.1002/advs.202405845 PMC11791960

[50] Devi-Marulkar P Fastenackels S Karapentiantz P Goc J Germain C Kaplon H . Regulatory T cells infiltrate the tumor-induced tertiary lymphoïd structures and are associated with poor clinical outcome in NSCLC. Commun Biol. (2022) 5:1416. doi: 10.1038/s42003-022-04356-y, PMID: 36566320 PMC9789959

[51] Yan Y Huang L Liu Y Yi M Chu Q Jiao D . Metabolic profiles of regulatory T cells and their adaptations to the tumor microenvironment: implications for antitumor immunity. J Hematol Oncol. (2022) 15:104. doi: 10.1186/s13045-022-01322-3, PMID: 35948909 PMC9364625

[52] Kim MJ Kim K Park HJ Kim G-R Hong KH Oh JH . Deletion of PD-1 destabilizes the lineage identity and metabolic fitness of tumor-infiltrating regulatory T cells. Nat Immunol. (2023) 24:148–61. doi: 10.1038/s41590-022-01373-1, PMID: 36577929

[53] Kumagai S Itahashi K Nishikawa H . Regulatory T cell-mediated immunosuppression orchestrated by cancer: towards an immuno-genomic paradigm for precision medicine. Nat Rev Clin Oncol. (2024) 21:337–53. doi: 10.1038/s41571-024-00870-6, PMID: 38424196

[54] Li Y Zhang C Jiang A Lin A Liu Z Cheng X . Potential anti-tumor effects of regulatory T cells in the tumor microenvironment: a review. J Transl Med. (2024) 22:293. doi: 10.1186/s12967-024-05104-y, PMID: 38509593 PMC10953261

[55] Wu Y Yi M Niu M Mei Q Wu K . Myeloid-derived suppressor cells: an emerging target for anticancer immunotherapy. Mol Cancer. (2022) 21:184. doi: 10.1186/s12943-022-01657-y, PMID: 36163047 PMC9513992

[56] Wang S Zhao X Wu S Cui D Xu Z . Myeloid-derived suppressor cells: key immunosuppressive regulators and therapeutic targets in hematological Malignancies. biomark Res. (2023) 11:34. doi: 10.1186/s40364-023-00475-8, PMID: 36978204 PMC10049909

[57] Lu J Luo Y Rao D Wang T Lei Z Chen X . Myeloid-derived suppressor cells in cancer: therapeutic targets to overcome tumor immune evasion. Exp Hematol Oncol. (2024) 13:39. doi: 10.1186/s40164-024-00505-7, PMID: 38609997 PMC11010322

[58] He S Zheng L Qi C . Myeloid-derived suppressor cells (MDSCs) in the tumor microenvironment and their targeting in cancer therapy. Mol Cancer. (2025) 24:5. doi: 10.1186/s12943-024-02208-3, PMID: 39780248 PMC11707952

[59] Blankenstein T Coulie PG Gilboa E Jaffee EM . The determinants of tumour immunogenicity. Nat Rev Cancer. (2012) 12:307–13. doi: 10.1038/nrc3246, PMID: 22378190 PMC3552609

[60] Huang J Yang B Peng Y Huang J Wong SHD Bian L . Nanomedicine-boosting tumor immunogenicity for enhanced immunotherapy. Adv Funct Mater. (2021) 31:2011171. doi: 10.1002/adfm.202011171

[61] Dogheim GM El Feel NE Abd El-Maksod EA Amer SS El-Gizawy SA Abd Elhamid AS . Nanomedicines as enhancers of tumor immunogenicity to augment cancer immunotherapy. Drug Discov Today. (2024) 29:103905. doi: 10.1016/j.drudis.2024.103905, PMID: 38295898

[62] Wang Z Wang Q Cao H Wang Z Wang D Liu J . Mitochondrial localized *in situ* self-assembly reprogramming tumor immune and metabolic microenvironment for enhanced cancer therapy. Adv Mater. (2024) 36:2311043. doi: 10.1002/adma.202311043, PMID: 38190762

[63] Wang Z Zhou P Li Y Zhang D Chu F Yuan F . A bimetallic polymerization network for effective increase in labile iron pool and robust activation of cGAS/STING induces ferroptosis-based tumor immunotherapy. Small. (2024) 20:2308397. doi: 10.1002/smll.202308397, PMID: 38072786

[64] Zhao C Wang C Shan W Wang Z Chen X Deng H . Nanomedicines for an enhanced immunogenic cell death-based *in situ* cancer vaccination response. Acc. Chem Res. (2024) 57:905–18. doi: 10.1021/acs.accounts.3c00771, PMID: 38417027

[65] Mikhail AS Morhard R Mauda-Havakuk M Kassin M Arrichiello A Wood BJ . Hydrogel drug delivery systems for minimally invasive local immunotherapy of cancer. Adv Drug Delivery Rev. (2023) 202:115083. doi: 10.1016/j.addr.2023.115083, PMID: 37673217 PMC11616795

[66] Pacheco C Baião A Ding T Cui W Sarmento B . Recent advances in long-acting drug delivery systems for anticancer drug. Adv Drug Delivery Rev. (2023) 194:114724. doi: 10.1016/j.addr.2023.114724, PMID: 36746307

[67] Shi P Cheng Z Zhao K Chen Y Zhang A Gan W . Active targeting schemes for nano-drug delivery systems in osteosarcoma therapeutics. J Nanobiotechnol. (2023) 21:103. doi: 10.1186/s12951-023-01826-1, PMID: 36944946 PMC10031984

[68] Al-Obaidy R Haider AJ Al-Musawi S Arsad N . Targeted delivery of paclitaxel drug using polymer-coated magnetic nanoparticles for fibrosarcoma therapy: *in vitro* and *in vivo* studies. Sci Rep. (2023) 13:3180. doi: 10.1038/s41598-023-30221-x, PMID: 36823237 PMC9950487

[69] Oh JY Choi E Jana B Go EM Jin E Jin S . Protein-precoated surface of metal-organic framework nanoparticles for targeted delivery. Small. (2023) 19:2300218. doi: 10.1002/smll.202300218, PMID: 36864579

[70] Xie S Sun W Fu T Liu X Chen P Qiu L . Aptamer-based targeted delivery of functional nucleic acids. J Am Chem Soc. (2023) 145:7677–91. doi: 10.1021/jacs.3c00841, PMID: 36987838

[71] Zhang J Gao B Ye B Sun Z Qian Z Yu L . Mitochondrial-targeted delivery of polyphenol-mediated antioxidases complexes against pyroptosis and inflammatory diseases. Adv Mater. (2023) 35:2208571. doi: 10.1002/adma.202208571, PMID: 36648306

[72] Zhu L Yu T Wang W Xu T Geng W Li N . Responsively degradable nanoarmor-assisted super resistance and stable colonization of probiotics for enhanced inflammation-targeted delivery. Adv Mater. (2024) 36:2308728. doi: 10.1002/adma.202308728, PMID: 38241751

[73] Chen X Wang S Chen Y Xin H Zhang S Wu D . Non-invasive activation of intratumoural gene editing for improved adoptive T-cell therapy in solid tumours. Nat Nanotechnol. (2023) 18:933–44. doi: 10.1038/s41565-023-01378-3, PMID: 37188968

[74] Maalej KM Merhi M Inchakalody VP Mestiri S Alam M Maccalli C . CAR-cell therapy in the era of solid tumor treatment: current challenges and emerging therapeutic advances. Mol Cancer. (2023) 22:20. doi: 10.1186/s12943-023-01723-z, PMID: 36717905 PMC9885707

[75] Chen T Wang M Chen Y Liu Y . Current challenges and therapeutic advances of CAR-T cell therapy for solid tumors. Cancer Cell Int. (2024) 24:133. doi: 10.1186/s12935-024-03315-3, PMID: 38622705 PMC11017638

[76] Long J Wang Y Jiang X Ge J Chen M Zheng B . Nanomaterials boost CAR-T therapy for solid tumors. Adv Healthcare Mater. (2024) 13:2304615. doi: 10.1002/adhm.202304615, PMID: 38483400

[77] Peng L Sferruzza G Yang L Zhou L Chen S . CAR-T and CAR-NK as cellular cancer immunotherapy for solid tumors, Cell. Mol Immunol. (2024) 21:1089–108. doi: 10.1038/s41423-024-01207-0, PMID: 39134804 PMC11442786

[78] Sun Q Li Y Shen W Shang W Y. xu J Chen J . Breaking-down tumoral physical barrier by remotely unwrapping metal-polyphenol-packaged hyaluronidase for optimizing photothermal/photodynamic therapy-induced immune response. Adv Mater. (2024) 36:2310673. doi: 10.1002/adma.202310673, PMID: 38284224

[79] Ni J-J Zhang Z-Z Ge M-J Chen J-Y Zhuo W . Immune-based combination therapy to convert immunologically cold tumors into hot tumors: an update and new insights. Acta Pharmacol Sin. (2023) 44:288–307. doi: 10.1038/s41401-022-00953-z, PMID: 35927312 PMC9889774

[80] Sun Q Hong Z Zhang C Wang L Han Z Ma D . Immune checkpoint therapy for solid tumours: clinical dilemmas and future trends. Signal Transduction Targeted Ther. (2023) 8:320. doi: 10.1038/s41392-023-01522-4, PMID: 37635168 PMC10460796

[81] Kaviyarasan V Das A Deka D Saha B Banerjee A Sharma NR . Advancements in immunotherapy for colorectal cancer treatment: a comprehensive review of strategies, challenges, and future prospective. Int J Colorectal Dis. (2024) 40:1. doi: 10.1007/s00384-024-04790-w, PMID: 39731596 PMC11682016

[82] Giurini EF Ralph O Pappas SG Gupta KH . Looking beyond checkpoint inhibitor monotherapy: uncovering new frontiers for pancreatic cancer immunotherapy. Mol Cancer Ther. (2025) 24:18–32. doi: 10.1158/1535-7163.MCT-24-0311, PMID: 39311547 PMC11694065

[83] He C Ding H Li L Chen J Mo X Ding Y . Gold nanoparticles enhance the ability of radiotherapy to induce immunogenic cell death in glioblastoma. Int J Nanomed. (2023) 18:5701–12. doi: 10.2147/IJN.S419712, PMID: 37841022 PMC10573392

[84] Liu T Pei P Shen W Hu L Yang K . Radiation-induced immunogenic cell death for cancer radioimmunotherapy. Small Methods. (2023) 7:2201401. doi: 10.1002/smtd.202201401, PMID: 36811166

[85] Galassi C Klapp V Yamazaki T Galluzzi L . Molecular determinants of immunogenic cell death elicited by radiation therapy, Immunol. Rev. (2024) 321:20–32., PMID: 37679959 10.1111/imr.13271 PMC11075037

[86] Wang P Zhao X Ying Y Huang S Liu T Zheng R . Programmed nanosystem for synergistic immunogenic cell death enhancing radiotherapy-mediated abscopal effect. Adv Funct Mater. (2025) 35:2412497. doi: 10.1002/adfm.202412497

[87] Sun Z Zhao M Wang W Hong L Wu Z Luo G . 5-ALA mediated photodynamic therapy with combined treatment improves anti-tumor efficacy of immunotherapy through boosting immunogenic cell death. Cancer Lett. (2023) 554:216032. doi: 10.1016/j.canlet.2022.216032, PMID: 36493899

[88] Zeng S Chen C Zhang L Liu X Qian M Cui H . Activation of pyroptosis by specific organelle-targeting photodynamic therapy to amplify immunogenic cell death for anti-tumor immunotherapy. Bioact. Mater. (2023) 25:580–93., PMID: 37056275 10.1016/j.bioactmat.2022.07.016 PMC10087757

[89] Liu X Lu Y Li X Luo L You J . Nanoplatform-enhanced photodynamic therapy for the induction of immunogenic cell death. J Controlled Release. (2024) 365:1058–73. doi: 10.1016/j.jconrel.2023.11.058, PMID: 38056695

[90] Cheng J Li J Wu J Kang W Feng K You Y . A novel hypocrellin B derivative for photodynamic therapy-driven cancer immunotherapy via triggering immunogenic cell death. Nano Today. (2025) 64:102809. doi: 10.1016/j.nantod.2025.102809

[91] Du F Chen Z Li X Hong X Wang L Cai F . Organelle-targeted photodynamic platforms: from ICD activation to tumor ablation, Chem. Commun. (2025) 61:12835–47. doi: 10.1039/D5CC03574K, PMID: 40762375

[92] Lee J-H Yang S-B Park SJ Kweon S Ma G Seo M . Cell-penetrating peptide like anti-programmed cell death-ligand 1 peptide conjugate-based self-assembled nanoparticles for immunogenic photodynamic therapy. ACS Nano. (2025) 19:2870–89. doi: 10.1021/acsnano.4c16128, PMID: 39761412

[93] Wu H Zhang Y Jiang L Huang H . Photodynamic therapy with photodegradable photosensitizers, Chem. Commun. (2025) 61:2627–35. 10.1039/d4cc05091f 39820656

[94] Yu J He X Wang Z Wang Y Liu S Li X . Combining PD-L1 inhibitors with immunogenic cell death triggered by chemo-photothermal therapy via a thermosensitive liposome system to stimulate tumor-specific immunological response. Nanoscale. (2021) 13:12966–78. doi: 10.1039/D1NR03288G, PMID: 34477780

[95] Xiang Q Yang C Luo Y Liu F Zheng J Liu W . Near-infrared II nanoadjuvant-mediated chemodynamic, photodynamic, and photothermal therapy combines immunogenic cell death with PD-L1 blockade to enhance antitumor immunity. Small. (2022) 18:2107809. doi: 10.1002/smll.202107809, PMID: 35143709

[96] Ran J Liu T Song C Wei Z Tang C Cao Z . Rhythm mild-temperature photothermal therapy enhancing immunogenic cell death response in oral squamous cell carcinoma, adv. Healthcare Mater. (2023) 12:2202360. doi: 10.1002/adhm.202202360, PMID: 36401600

[97] Wang J Ma J Tai Z Li L Zhang T Cheng T . Nanocarrier-mediated immunogenic cell death for melanoma treatment. Int J Nanomed. (2023) 18:7149–72. 10.2147/IJN.S434582 PMC10697015 38059000

[98] Peng J Li S Ti H . Sensitize tumor immunotherapy: immunogenic cell death inducing nanosystems. Int J Nanomed. (2024) 19:5895–930., PMID: 38895146 10.2147/IJN.S457782 PMC11184231

[99] Yu SH Yoon I Kim Y-J . Ex vivo photothermal treatment-induced immunogenic cell death for anticancer vaccine development. Int Immunopharmacol. (2024) 127:111450., PMID: 38157695 10.1016/j.intimp.2023.111450

[100] Zhang G Wang N Sun H Fu X Zhai S Cui J . Self-adjuvanting photosensitizer nanoparticles for combination photodynamic immunotherapy. Biomater. Sci. (2021) 9:6940–9., PMID: 34528658 10.1039/d1bm01139a

[101] Li J Dai J Zhuang Z Meng Z Hu J-J Lou X . Combining PD-L1 blockade with immunogenic cell death induced by AIE photosensitizer to improve antitumor immunity. Biomaterials. (2022) 291:121899. doi: 10.1016/j.biomaterials.2022.121899, PMID: 36343606

[102] Li Z Lai X Fu S Ren L Cai H Zhang H . Immunogenic cell death activates the tumor immune microenvironment to boost the immunotherapy efficiency. Adv Sci. (2022) 9:2201734. doi: 10.1002/advs.202201734, PMID: 35652198 PMC9353475

[103] Huang H Xie W Hu D He X Li R Zhang X . Type I photodynamic therapy with a self-degradable conjugated polyelectrolyte in combination with CpG adjuvant for cancer immunotherapy. Chem Eng. J. (2023) 451:138617.

[104] He L Wang J Zhu P Chen J Zhao S Liu X . Intelligent manganese dioxide nanocomposites induce tumor immunogenic cell death and remould tumor microenvironment. Chem Eng. J. (2023) 461:141369.

[105] Kong R-J Li Y-M Huang J-Q Yan N Wu Y-Y Cheng H . Self-delivery photodynamic re-educator enhanced tumor treatment by inducing immunogenic cell death and improving immunosuppressive microenvironments, ACS appl. Mater Interfaces. (2023) 15:59165–74. doi: 10.1021/acsami.3c13188, PMID: 38100370

[106] Yuan H Ma J Huang W Gong P Shi F Xu X . Antitumor effects of a distinct sonodynamic nanosystem through enhanced induction of immunogenic cell death and ferroptosis with modulation of tumor microenvironment. JACS Au. (2023) 3:1507–20. doi: 10.1021/jacsau.3c00156, PMID: 37234112 PMC10206594

[107] Ma J Yuan H Zhang J Sun X Yi L Li W . An ultrasound-activated nanoplatform remodels tumor microenvironment through diverse cell death induction for improved immunotherapy, J. Controlled Release. (2024) 370:501–15. doi: 10.1016/j.jconrel.2024.05.001, PMID: 38703950

[108] Ding R Yang H Wang J Liu Y Mu S Wang D . Advances in stimuli-responsive release strategies for sonosensitizers in synergistic sonodynamic immunotherapy against tumors. Adv Healthcare Mater. (2025), 2502183. doi: 10.1002/adhm.202502183, PMID: 40665890

[109] Yu L Gao L Liang B Zhang L Wu M Liu J . Polymer-based nanodrugs enhance sonodynamic therapy through epigenetic reprogramming of the immunosuppressive tumor microenvironment, J. Controlled Release. (2025) 380:125–37. doi: 10.1016/j.jconrel.2025.01.086, PMID: 39892649

[110] Feng L Qian C Zhao Y . Recent advances in covalent organic framework-based nanosystems for bioimaging and therapeutic applications, ACS mater. Lett. (2020) 2:1074–92.

[111] Guan Q Zhou L-L Li W-Y Li Y-A Dong Y-B . Covalent organic frameworks (COFs) for cancer therapeutics, chem. Eur J. (2020) 26:5583–91. doi: 10.1002/chem.201905150, PMID: 31880368

[112] Zhu Y Xin N Qiao Z Chen S Zeng L Zhang Y . Bioactive MOFs based theranostic agent for highly effective combination of multimodal imaging and chemo-phototherapy. Adv Healthcare Mater. (2020) 9:2000205. doi: 10.1002/adhm.202000205, PMID: 32548979

[113] Yan J-H Shao K Wu L-j Hu J Man M-M Li X-q . Upconversion-nanoparticle-based smart drug-delivery platforms for multimodal imaging-guided cancer therapies, ACS appl. Nano Mater. (2022) 5:15473–87.

[114] Rahmani Khalili N Banitalebi Dehkordi A Amiri A Mohammadi Ziarani G Badiei A Cool P . Tailored covalent organic framework platform: from multistimuli, targeted dual drug delivery by architecturally engineering to enhance photothermal tumor therapy. ACS Appl Mater Interfaces. (2024) 16:28245–62. doi: 10.1021/acsami.4c05989, PMID: 38770930

[115] Zhou L-L Guan Q Dong Y-B . Covalent organic frameworks: opportunities for rational materials design in cancer therapy, angew. Chem. Int Ed. (2024) 63:e202314763., PMID: 37983842 10.1002/anie.202314763

[116] Guo J-C Feng Q-H Yang S Du J Zhou X Zhou S-H . Multifunctional drug delivery nanoparticles based on fe-containing metal–organic framework for fluorescence image-guided combined chemical/chemodynamic/photodynamic colorectal cancer treatment. ACS Appl Bio Mater. (2025) 8:8160–71. doi: 10.1021/acsabm.5c01102, PMID: 40813300

[117] Cai Z Xin F Wei Z Wu M Lin X Du X . Photodynamic therapy combined with antihypoxic signaling and cpG adjuvant as an *in situ* tumor vaccine based on metal–organic framework nanoparticles to boost cancer immunotherapy. Adv Healthcare Mater. (2020) 9:1900996. doi: 10.1002/adhm.201900996, PMID: 31746153

[118] Dai J Wu M Wang Q Ding S Dong X Xue L . Red blood cell membrane-camouflaged nanoparticles loaded with AIEgen and Poly(I : C) for enhanced tumoral photodynamic-immunotherapy. Natl Sci Rev. (2021) 8:nwab039. doi: 10.1093/nsr/nwab039, PMID: 34691671 PMC8288176

[119] Fang L Zhao Z Wang J Xiao P Sun X Ding Y . Light-controllable charge-reversal nanoparticles with polyinosinic-polycytidylic acid for enhancing immunotherapy of triple negative breast cancer. Acta Pharm Sin B. (2022) 12:353–63. doi: 10.1016/j.apsb.2021.06.006, PMID: 35127391 PMC8800000

[120] Lei H Kim JH Son S Chen L Pei Z Yang Y . Immunosonodynamic therapy designed with activatable sonosensitizer and immune stimulant imiquimod. ACS Nano. (2022) 16:10979–93. doi: 10.1021/acsnano.2c03395, PMID: 35723442

[121] Yu G Dong F Ge W Sun L Zhang L Yuan L . Self-assembled nanospheres mediate phototherapy and deliver CpG oligodeoxynucleotides to enhance cancer immunotherapy of breast cancer and melanoma. Nano Today. (2022) 44:101498. doi: 10.1016/j.nantod.2022.101498

[122] Liu D Fu L Gong L Li S Li K Liu K . Proton-gradient-driven porphyrin-based liposome remote-loaded with imiquimod as *in situ* nanoadjuvants for synergistically augmented tumor photoimmunotherapy. ACS Appl Mater Interfaces. (2024) 16:8403–16. doi: 10.1021/acsami.3c17133, PMID: 38334116

[123] Sun Z Wang J Guo B Zhao S Miao S Xia M . Nano-golden adjuvant-polymersomes empower tumor photothermal-immunotherapy. J Controlled Release. (2025) 385:113976. doi: 10.1016/j.jconrel.2025.113976, PMID: 40545199

[124] Bagheri AR Li C Zhang X Zhou X Aramesh N Zhou H . Recent advances in covalent organic frameworks for cancer diagnosis and therapy, Biomater. Sci. (2021) 9:5745–61., PMID: 34318797 10.1039/d1bm00960e

[125] He X Jiang Z Akakuru OU Li J Wu A . Nanoscale covalent organic frameworks: from controlled synthesis to cancer therapy. Chem Commun. (2021) 57:12417–35. doi: 10.1039/D1CC04846E, PMID: 34734601

[126] Duan Y Yu Y Liu P Gao Y Dai X Zhang L . Reticular chemistry-enabled sonodynamic activity of covalent organic frameworks for nanodynamic cancer therapy. Angew. Chem. Int Ed. (2023) 62:e202302146., PMID: 36894504 10.1002/anie.202302146

[127] Ghosh P Banerjee P . Drug delivery using biocompatible covalent organic frameworks (COFs) towards a therapeutic approach. Chem Commun. (2023) 59:12527–47. doi: 10.1039/D3CC01829F, PMID: 37724444

[128] Khan N Slathia G Kaliya K Saneja A . Recent progress in covalent organic frameworks for cancer therapy. Drug Discov Today. (2023) 28:103602. doi: 10.1016/j.drudis.2023.103602, PMID: 37119962

[129] Li W-Y Wan J-J Kan J-L Wang B Song T Guan Q . A biodegradable covalent organic framework for synergistic tumor therapy. Chem Sci. (2023) 14:1453–60. doi: 10.1039/D2SC05732H, PMID: 36794183 PMC9906711

[130] Guo J Kong S Lian Y Zhao M . Recent bio-applications of covalent organic framework-based nanomaterials. Chem Commun. (2024) 60:918–34. doi: 10.1039/D3CC04368A, PMID: 38168699

[131] Singh N Won M Xu Y Yoon C Yoo J Li M . Covalent organic framework nanoparticles: Overcoming the challenges of hypoxia in cancer therapy. Coord Chem Rev. (2024) 499:215481. doi: 10.1016/j.ccr.2023.215481

[132] Diercks CS Yaghi OM . The atom, the molecule, and the covalent organic framework. Science. (2017) 355:eaal1585. doi: 10.1126/science.aal1585, PMID: 28254887

[133] Huang N Wang P Jiang D . Covalent organic frameworks: a materials platform for structural and functional designs. Nat Rev Mater. (2016) 1:16068. doi: 10.1038/natrevmats.2016.68

[134] Segura JL Mancheño MJ Zamora F . Covalent organic frameworks based on Schiff-base chemistry: synthesis, properties and potential applications, Chem. Soc Rev. (2016) 45:5635–71. doi: 10.1039/C5CS00878F, PMID: 27341661

[135] Guan X Chen F Fang Q Qiu S . Design and applications of three dimensional covalent organic frameworks, Chem. Soc Rev. (2020) 49:1357–84. doi: 10.1039/C9CS00911F, PMID: 32067000

[136] Qian C Feng L Teo WL Liu J Zhou W Wang D . Imine and imine-derived linkages in two-dimensional covalent organic frameworks, Nat. Rev Chem. (2022) 6:881–98., PMID: 37117702 10.1038/s41570-022-00437-y

[137] Liang R-R Jiang S-Y R.-H. A Zhao X . Two-dimensional covalent organic frameworks with hierarchical porosity. Chem Soc Rev. (2020) 49:3920–51. doi: 10.1039/D0CS00049C, PMID: 32427238

[138] Guan X Chen F Qiu S Fang Q . Three-dimensional covalent organic frameworks: from synthesis to applications. Angew. Chem. Int Ed. (2023) 62:e202213203. 10.1002/anie.202213203 36253336

[139] Xue S Ma X Wang Y Duan G Zhang C Liu K . Advanced development of three-dimensional covalent organic frameworks: Valency design, functionalization, and applications. Coord Chem Rev. (2024) 504:215659. doi: 10.1016/j.ccr.2024.215659

[140] Hao K Lin L Sun P Hu Y Atsushi M Guo Z . Cationic flexible organic framework for combination of photodynamic therapy and genetic immunotherapy against tumors. Small. (2021) 17:2008125. doi: 10.1002/smll.202008125, PMID: 33760358

[141] Zhang L Song A Yang Q-C Li S-J Wang S Wan S-C . Integration of AIEgens into covalent organic frameworks for pyroptosis and ferroptosis primed cancer immunotherapy. Nat Commun. (2023) 14:5355. doi: 10.1038/s41467-023-41121-z, PMID: 37660063 PMC10475094

[142] Zhou Q Huang G Si J Wu Y Jin S Ji Y . Potent covalent organic framework nanophotosensitizers with staggered type I/II motifs for photodynamic immunotherapy of hypoxic tumors. ACS Nano. (2024) 18:35671–83. doi: 10.1021/acsnano.4c14555, PMID: 39698912

[143] Yan T Liao Q Chen Z Xu Y Zhu W Hu P . β-Ketoenamine covalent organic framework nanoplatform combined with immune checkpoint blockade via photodynamic immunotherapy inhibit glioblastoma progression. Bioact. Mater. (2025) 44:531–43., PMID: 39584065 10.1016/j.bioactmat.2024.10.029 PMC11583667

[144] Guan Q Fu D-D Li Y-A Kong X-M Wei Z-Y Li W-Y . BODIPY-decorated nanoscale covalent organic frameworks for photodynamic therapy. iScience. (2019) 14:180–98. doi: 10.1016/j.isci.2019.03.028, PMID: 30981114 PMC6461589

[145] Zhang L Wang S Zhou Y Wang C Zhang X-Z Deng H . Covalent organic frameworks as favorable constructs for photodynamic therapy. Angew. Chem. Int Ed. (2019) 58:14213–8. doi: 10.1002/anie.201909020, PMID: 31347259

[146] Chen S Sun T Zheng M Xie Z . Carbon dots based nanoscale covalent organic frameworks for photodynamic therapy. Adv Funct Mater. (2020) 30:2004680. doi: 10.1002/adfm.202004680

[147] Wang S-B Chen Z-X Gao F Zhang C Zou M-Z Ye J-J . Remodeling extracellular matrix based on functional covalent organic framework to enhance tumor photodynamic therapy. Biomaterials. (2020) 234:119772. doi: 10.1016/j.biomaterials.2020.119772, PMID: 31945618

[148] Liang S Li M-H Qi M-L Hui H Zhang H-P Zhou J . Reactive oxygen species-responsive pillararene-embedded covalent organic frameworks with amplified antimicrobial photodynamic therapy for the targeted elimination of periodontitis pathogens, *Nano lett*. (2024) 24:13708–17. doi: 10.1021/acs.nanolett.4c03788, PMID: 39417607

[149] Zhang X Dou Y Liu S Chen P Wen Y Li J . Rationally designed benzobisthiadiazole-based covalent organic framework for high-performance NIR-II fluorescence imaging-guided photodynamic therapy. Adv Healthcare Mater. (2024) 13:2303842. doi: 10.1002/adhm.202303842, PMID: 38458147

[150] Zhen W Kang DW Fan Y Wang Z Germanas T Nash GT . Simultaneous protonation and metalation of a porphyrin covalent organic framework enhance photodynamic therapy. J Am Chem Soc. (2024) 146:16609–18. doi: 10.1021/jacs.4c03519, PMID: 38837955

[151] Chen M Li H Liu C Liu J Feng Y Wee AGH . Porphyrin- and porphyrinoid-based covalent organic frameworks (COFs): From design, synthesis to applications. Coord Chem Rev. (2021) 435:213778. doi: 10.1016/j.ccr.2021.213778

[152] Chen R Wang Y Ma Y Mal A Gao X-Y Gao L . Rational design of isostructural 2D porphyrin-based covalent organic frameworks for tunable photocatalytic hydrogen evolution. Nat Commun. (2021) 12:1354. doi: 10.1038/s41467-021-21527-3, PMID: 33649344 PMC7921403

[153] Harvey PD . Porphyrin-based metal- and covalent-organic frameworks as heterogeneous nanosized photocatalysts in organic synthesis. J Mater Chem C. (2021) 9:16885–910. doi: 10.1039/D1TC04147A

[154] Li X-G Li J Chen J Rao L Zheng L Yu F . Porphyrin-based covalent organic frameworks from design, synthesis to biological applications. Biomater. Sci. (2024) 12:2766–85. 10.1039/d4bm00214h 38717456

[155] Zhang L Xiao Y Yang Q-C Yang L-L Wan S-C Wang S . Staggered stacking covalent organic frameworks for boosting cancer immunotherapy. Adv Funct Mater. (2022) 32:2201542. doi: 10.1002/adfm.202201542

[156] Zhang L Wang S Yang Q-C Song A Wang Y-Y Wang W-D . Radioactive diselenide bonded covalent organic framework. Adv Mater. (2025), 2413002. doi: 10.1002/adma.202413002, PMID: 39988842

[157] Liu D Liang M Tao Y Liu H Liu Q Bing W . Hypoxia-accelerating pyroptosis nanoinducers for promoting image-guided cancer immunotherapy. Biomaterials. (2024) 309:122610. doi: 10.1016/j.biomaterials.2024.122610, PMID: 38749307

[158] Gan S Tong X Zhang Y Wu J Hu Y Yuan A . Covalent organic framework-supported molecularly dispersed near-infrared dyes boost immunogenic phototherapy against tumors. Adv Funct Mater. (2019) 29:1902757. doi: 10.1002/adfm.201902757

[159] Wang W Yu Y Jin Y Liu X Shang M Zheng X . Two-dimensional metal-organic frameworks: from synthesis to bioapplications, J. Nanobiotechnol. (2022) 20:207. doi: 10.1186/s12951-022-01395-9, PMID: 35501794 PMC9059454

[160] Zhang B Su X Kang J Chen L . Covalent organic frameworks as multifunctional nanoplatforms for precise cancer therapy: design principles, applications, and future perspectives. Coord Chem Rev. (2026) 549:217250. doi: 10.1016/j.ccr.2025.217250

[161] Vitale I Shema E Loi S Galluzzi L . Intratumoral heterogeneity in cancer progression and response to immunotherapy. Nat Med. (2021) 27:212–24. doi: 10.1038/s41591-021-01233-9, PMID: 33574607

[162] Jia Q Wang A Yuan Y Zhu B Long H . Heterogeneity of the tumor immune microenvironment and its clinical relevance. Exp Hematol Oncol. (2022) 11:24. doi: 10.1186/s40164-022-00277-y, PMID: 35461288 PMC9034473

[163] Tian Y Xie D Yang L . Engineering strategies to enhance oncolytic viruses in cancer immunotherapy. Signal Transduction Targeted Ther. (2022) 7:117. doi: 10.1038/s41392-022-00951-x, PMID: 35387984 PMC8987060

[164] Lee D Huntoon K Lux J Kim BYS Jiang W . Engineering nanomaterial physical characteristics for cancer immunotherapy. Nat Rev Bioeng. (2023) 1:499–517.

[165] Ou W Stewart S White A Kwizera EA Xu J Fang Y . *In-situ* cryo-immune engineering of tumor microenvironment with cold-responsive nanotechnology for cancer immunotherapy. Nat Commun. (2023) 14:392. doi: 10.1038/s41467-023-36045-7, PMID: 36693842 PMC9873931

[166] Lu Z Bai S Jiang Y Wu S Xu D Chen Y . Porphyrin-based covalent organic framework for imaging-guided cancer combinatorial immuno-sonodynamic therapy. Adv Funct Mater. (2022) 32:2207749. doi: 10.1002/adfm.202207749

[167] Zhang L Yang Q-C Wang S Xiao Y Wan S-C Deng H . Engineering multienzyme-mimicking covalent organic frameworks as pyroptosis inducers for boosting antitumor immunity. Adv Mater. (2022) 34:2108174. doi: 10.1002/adma.202108174, PMID: 34918837

[168] Zhang L Zhang B Zhang M-J Li W Li H Jiao Y . Trigger inducible tertiary lymphoid structure formation using covalent organic frameworks for cancer immunotherapy. Nat Commun. (2025) 16:44. doi: 10.1038/s41467-024-55430-4, PMID: 39747845 PMC11696883

[169] Sun M Liu Z Wu L Yang J Ren J Qu X . Bioorthogonal-activated *in situ* vaccine mediated by a COF-based catalytic platform for potent cancer immunotherapy. J Am Chem Soc. (2023) 145:5330–41. doi: 10.1021/jacs.2c13010, PMID: 36815731

[170] Yang Q-C Wang Y-Y Wang S Song A Wang W-D Zhang L . Engineered bacterial membrane biomimetic covalent organic framework as nano-immunopotentiator for cancer immunotherapy. Bioact. Mater. (2025) 47:283–94., PMID: 39925708 10.1016/j.bioactmat.2025.01.018 PMC11803166

[171] Chen G Gu X Xue J Zhang X Yu X Zhang Y . Effects of neoadjuvant stereotactic body radiotherapy plus adebrelimab and chemotherapy for triple-negative breast cancer: A pilot study. eLife. (2023) 12:e91737. doi: 10.7554/eLife.91737.sa2, PMID: 38131294 PMC10746137

[172] Zhang C Wang P Y.n. Zhang P Huang X Wang Y Ran L . Biodegradable nanoplatform upregulates tumor microenvironment acidity for enhanced cancer therapy via synergistic induction of apoptosis, ferroptosis, and anti-angiogenesis. J Nanobiotechnol. (2023) 21:59. doi: 10.1186/s12951-023-01814-5, PMID: 36810074 PMC9945394

[173] Li W Xin H Gao W Yuan P Ni F Ma J . NIR-IIb fluorescence antiangiogenesis copper nano-reaper for enhanced synergistic cancer therapy. J Nanobiotechnol. (2024) 22:73. doi: 10.1186/s12951-024-02343-5, PMID: 38374027 PMC10877799

[174] Ping Y Yae H Quansheng Z . The CXCL12-CXCR4 signaling axis plays a key role in cancer metastasis and is a potential target for developing novel therapeutics against metastatic cancer. Curr Med Chem. (2020) 27:5543–61. doi: 10.2174/0929867326666191113113110, PMID: 31724498

[175] Wang N Zhang Y Liu H Wang A Ren T Gou J . Toxicity reduction and efficacy promotion of doxorubicin in the treatment of breast tumors assisted by enhanced oral absorption of curcumin-loaded lipid–polyester mixed nanoparticles. Mol Pharmaceutics. (2020) 17:4533–47. doi: 10.1021/acs.molpharmaceut.0c00718, PMID: 33201717

[176] Liu C Yang M Zhang D Chen M Zhu D . Clinical cancer immunotherapy: Current progress and prospects. Front Immunol. (2022) 13:2022. doi: 10.3389/fimmu.2022.961805, PMID: 36304470 PMC9592930

[177] Rui R Zhou L He S . Cancer immunotherapies: advances and bottlenecks. Front Immunol. (2023) 14:2023. doi: 10.3389/fimmu.2023.1212476, PMID: 37691932 PMC10484345

[178] Akinsipe T Mohamedelhassan R Akinpelu A Pondugula SR Mistriotis P Avila LA . Cellular interactions in tumor microenvironment during breast cancer progression: new frontiers and implications for novel therapeutics. Front Immunol. (2024) 15:2024. doi: 10.3389/fimmu.2024.1302587, PMID: 38533507 PMC10963559

[179] Zheng R Liu X Zhang Y Liu Y Wang Y Guo S . Frontiers and future of immunotherapy for pancreatic cancer: from molecular mechanisms to clinical application, Front. Immunol. (2024) 15:2024. doi: 10.3389/fimmu.2024.1383978, PMID: 38756774 PMC11096556

[180] Liang D-M Li Y-J Zhang J-X Shen H-H Wu C-X Xie N . m6A-methylated KCTD21-AS1 regulates macrophage phagocytosis through CD47 and cell autophagy through TIPR. Commun Biol. (2024) 7:215. doi: 10.1038/s42003-024-05854-x, PMID: 38383737 PMC10881998

[181] Miao YD Quan WX Tang XL Shi WW Li Q Li RJ . Uncovering the flip side of immune checkpoint inhibitors: a comprehensive review of immune-related adverse events and predictive biomarkers. Int J Biol Sci. (2024) 20:621–42. doi: 10.7150/ijbs.89376, PMID: 38169638 PMC10758091

[182] Yue L Geng F Jin J Li W Liu B Du M . Lactobacillus reuteri assists engineered bacteria that target tumors to release PD-L1nb to mitigate the adverse effects of breast cancer immunotherapy. Biotechnol J. (2024) 19:e202400428. doi: 10.1002/biot.202400428, PMID: 39711089

[183] Chen G Zhao Y Xu Y Zhu C Liu T Wang K . Chitosan nanoparticles for oral photothermally enhanced photodynamic therapy of colon cancer. Int J Pharm. (2020) 589:119763. doi: 10.1016/j.ijpharm.2020.119763, PMID: 32898629

[184] Chen J Wang Z Wang W Ren S Xue J Zhong L . SYT16 is a prognostic biomarker and correlated with immune infiltrates in glioma: A study based on TCGA data. Int Immunopharmacol. (2020) 84:106490. doi: 10.1016/j.intimp.2020.106490, PMID: 32289666

[185] Yong L Ji L Jianqiang Q Chi M Jing G Weijie G . Recent advances in multi-target drugs targeting protein kinases and histone deacetylases in cancer therapy. Curr Med Chem. (2020) 27:7264–88. doi: 10.2174/0929867327666200102115720, PMID: 31894740

[186] Karaosmanoglu S Zhou M Shi B Zhang X Williams GR Chen X . Carrier-free nanodrugs for safe and effective cancer treatment. J Controlled Release. (2021) 329:805–32. doi: 10.1016/j.jconrel.2020.10.014, PMID: 33045313

[187] Fan Z Wu C Chen M Jiang Y Wu Y Mao R . The generation of PD-L1 and PD-L2 in cancer cells: From nuclear chromatin reorganization to extracellular presentation. Acta Pharm Sin B. (2022) 12:1041–53. doi: 10.1016/j.apsb.2021.09.010, PMID: 35530130 PMC9069407

[188] Zhang Q Wang X Chen J Wu J Zhou M Xia R . Recent progress of porphyrin metal–organic frameworks for combined photodynamic therapy and hypoxia-activated chemotherapy. Chem Commun. (2024) 60:13641–52. doi: 10.1039/D4CC04512B, PMID: 39497649

[189] Zou Y Chen J Luo X Qu Y Zhou M Xia R . Porphyrin-engineered nanoscale metal-organic frameworks: enhancing photodynamic therapy and ferroptosis in oncology, Front. Pharmacol. (2024) 15: 2024. doi: 10.3389/fphar.2024.1481168, PMID: 39512824 PMC11541831

[190] Qi C Chen M Yuan Z Wang W Zheng X . Phototherapeutic antibacterial applications of porphyrin-based metal–organic frameworks, Chem. Commun. (2025) 61:15313–28. doi: 10.1039/D5CC04455C, PMID: 40928219

[191] Qi C Chen J Qu Y Luo X Wang W Zheng X . Recent advances in porphyrin-based covalent organic frameworks for synergistic photodynamic and photothermal therapy. Pharmaceutics. (2024) 16:1625. doi: 10.3390/pharmaceutics16121625, PMID: 39771603 PMC11678282

[192] Zhang X Wang S Tang K Pan W Xu H Li Y . Cu2+ Embedded three-dimensional covalent organic framework for multiple ROS-based cancer immunotherapy, ACS appl. Mater Interfaces. (2022) 14:30618–25. doi: 10.1021/acsami.2c07739, PMID: 35763788

[193] Liu S Zhou Y Hu C Cai L Pang M . Covalent organic framework-based nanocomposite for synergetic photo-, chemodynamic-, and immunotherapies, ACS appl. Mater Interfaces. (2020) 12:43456–65. doi: 10.1021/acsami.0c12824, PMID: 32880166

[194] Yang L-L Zhang L Wan S-C Wang S Wu Z-Z Yang Q-C . Two-photon absorption induced cancer immunotherapy using covalent organic frameworks. Adv Funct Mater. (2021) 31:2103056. doi: 10.1002/adfm.202103056

[195] Wang D Lin L Li T Meng M Hao K Guo Z . Etching bulk covalent organic frameworks into nanoparticles of uniform and controllable size by the molecular exchange etching method for sonodynamic and immune combination antitumor therapy. Adv Mater. (2022) 34:2205924. doi: 10.1002/adma.202205924, PMID: 36039617

[196] Wang D Li T Lin L Meng M Hao K Guo Z . Magnetic covalent organic framework-based nanoadjuvant for multi-amplify sonodynamic antitumor therapy effect. Nano Today. (2024) 54:102088. doi: 10.1016/j.nantod.2023.102088

[197] Tai Y Chen Z Luo T Luo B Deng C Lu Z . MOF@COF nanocapsules enhance soft tissue sarcoma treatment: synergistic effects of photodynamic therapy and PARP inhibition on tumor growth suppression and immune response activation. Adv Healthcare Mater. (2024) 13:2303911. doi: 10.1002/adhm.202303911, PMID: 38215731

[198] Shi Y Guo Z Fu Q Shen X Zhang Z Sun W . Localized nuclear reaction breaks boron drug capsules loaded with immune adjuvants for cancer immunotherapy. Nat Commun. (2023) 14:1884. doi: 10.1038/s41467-023-37253-x, PMID: 37019890 PMC10076324

[199] Lin Z Zou S Wen K . The crosstalk of CD8+ T cells and ferroptosis in cancer. Front Immunol. (2024) 14:2023. doi: 10.3389/fimmu.2023.1255443, PMID: 38288118 PMC10822999

[200] Wang W Li H Liang S Hu Y Ding J Wu X . Bridging the gap: ferroptosis of immune cells in the tumor microenvironment. Front Immunol. (2025) 16:2025. doi: 10.3389/fimmu.2025.1648432, PMID: 41035634 PMC12479322

[201] Xia C Luo Z Feng Z Zhang Q Xia C . Mechanism and application of copper-based nanomedicines in activating tumor immunity through oxidative stress modulation. Front Pharmacol. (2025) 16: 2025. doi: 10.3389/fphar.2025.1646890, PMID: 40717985 PMC12289571

[202] Xia W Lv Y Zou Y Kang Z Li Z Tian J . The role of ferroptosis in colorectal cancer and its potential synergy with immunotherapy, Front. Immunol Volume. (2025) 15: 2024., PMID: 39850905 10.3389/fimmu.2024.1526749 PMC11754392

[203] Zou Z Chang H Li H Wang S . Induction of reactive oxygen species: an emerging approach for cancer therapy. Apoptosis. (2017) 22:1321–35. doi: 10.1007/s10495-017-1424-9, PMID: 28936716

[204] Cun J-E Pan Y Zhang Z Lu Y Li J Pan Q . Photo-enhanced upcycling H2O2 into hydroxyl radicals by IR780-embedded Fe3O4 @ MIL-100 for intense nanocatalytic tumor therapy. Biomaterials. (2022) 287:121687. doi: 10.1016/j.biomaterials.2022.121687, PMID: 35872555

[205] Ma G Liu Z Zhu C Chen H Kwok RTK Zhang P . H2O2-responsive NIR-II AIE nanobomb for carbon monoxide boosting low-temperature photothermal therapy, angew. Chem. Int Ed. (2022) 61:e202207213. 10.1002/anie.202207213 35838004

[206] Glorieux C Liu S Trachootham D Huang P . Targeting ROS in cancer: rationale and strategies. Nat Rev Drug Discov. (2024) 23:583–606. doi: 10.1038/s41573-024-00979-4, PMID: 38982305

[207] Gong B Zhang Q Chen J Qu Y Luo X Wang W . Recent advances in glutathione depletion-enhanced porphyrin-based nMOFs for photodynamic therapy. Pharmaceutics. (2025) 17:244. doi: 10.3390/pharmaceutics17020244, PMID: 40006611 PMC11860060

[208] Yang S Gaohua H Quan C Lei Y Peng W Qi Z . Au-pt nanoparticle formulation as a radiosensitizer for radiotherapy with dual effects. Int J Nanomed. (2021) 16:239–48. doi: 10.2147/IJN.S287523, PMID: 33469284 PMC7811476

[209] Ye Z Liu J Liu Y Zhao Y Li Z Xu B . Hybrid nanopotentiators with dual cascade amplification for glioma combined interventional therapy. J Controlled Release. (2024) 372:95–112. doi: 10.1016/j.jconrel.2024.06.016, PMID: 38851536

[210] Gong B Zhang Q Qu Y Zheng X Wang W . Nanoscale porphyrin-based metal–organic frameworks for enhanced radiotherapy–radiodynamic therapy: A comprehensive review. Pharmaceutics. (2025) 17:883. doi: 10.3390/pharmaceutics17070883, PMID: 40733092 PMC12299130

[211] Xiong S Song D Xiang Y Li Y Zhong Y Li H . Reactive oxygen species, not Ca2+, mediates methotrexate-induced autophagy and apoptosis in spermatocyte cell line. Basic Clin Pharmacol Toxicol. (2020) 126:144–52. doi: 10.1111/bcpt.13306, PMID: 31420979

[212] Cheng Q Chen J Guo H Lu J-L Zhou J Guo X-Y . Pyrroloquinoline quinone promotes mitochondrial biogenesis in rotenone-induced Parkinson’s disease model via AMPK activation. Acta Pharmacol Sin. (2021) 42:665–78. doi: 10.1038/s41401-020-0487-2, PMID: 32860006 PMC8115282

[213] Hu Y Li D Wei H Zhou S Chen W Yan X . Neurite extension and orientation of spiral ganglion neurons can be directed by superparamagnetic iron oxide nanoparticles in a magnetic field. Int J Nanomed. (2021) 16:4515–26. doi: 10.2147/IJN.S313673, PMID: 34239302 PMC8259836

[214] Wei H Yangnan H Junguo W Xia G Xiaoyun Q Tang M . Superparamagnetic iron oxide nanoparticles: cytotoxicity, metabolism, and cellular behavior in biomedicine applications, it. J Nanomed. (2021) 16:6097–113. doi: 10.2147/IJN.S321984, PMID: 34511908 PMC8418330

